# Duration of forensic psychiatric care and subsequent criminal recidivism in individuals sentenced in Sweden between 2009 and 2019

**DOI:** 10.3389/fpsyt.2023.1129993

**Published:** 2023-03-14

**Authors:** Lenka Sivak, Jonas Forsman, Thomas Masterman

**Affiliations:** ^1^Department of Clinical Neuroscience, Karolinska Institute, Stockholm, Sweden; ^2^Department of Forensic Psychiatry, National Board of Forensic Medicine, Huddinge, Sweden

**Keywords:** forensic psychiatry, Sweden, criminal recidivism, violent crime, treatment duration, criminal justice, register, Kaplan–Meier estimate

## Abstract

**Background:**

The duration of forensic psychiatric care is in Sweden not determined at the time of sentencing; instead, offenders are regularly evaluated, often with regard to risk of criminal recidivism. The length and justifiability of such a sanction have been greatly debated; however, previous estimates of treatment duration based on datasets delimited to discharged patients—have provided an uncertain groundwork for these deliberations. The aim of this study was to use a more suitable approach to calculate average duration of forensic psychiatric care and to examine the relationship between length of treatment and subsequent recidivism after discharge.

**Methods:**

This retrospective cohort study focused on offenders sentenced to forensic psychiatric care in Sweden between 2009 and 2019 and registered in the Swedish National Forensic Psychiatric Register (*n* = 2064), with a follow-up period until May 2020. We used Kaplan–Meier estimator to calculate and visualize treatment duration including analyses comparing levels of relevant variables, and then evaluated criminal recidivism in patients discharged from treatment between 2009 and 2019 (*n* = 640), after stratification for the same variables and dichotomization by treatment duration.

**Results:**

The median duration of forensic psychiatric care was estimated to 89.7 months (95% CI 83.2–95.8). Treatment was longer in offenders who committed violent crimes, suffered from psychosis, or had a history of substance use disorder, and in offenders whose sentences included special court supervision. The cumulative incidence of recidivism in patients discharged from treatment was estimated to 13.5% at 12 months (95% CI 10.6–16.2) and 19.5% at 24 months (95% CI 16.0–22.8). Corresponding cumulative incidence of violent crime post discharge was 6.3% at 12 months (95% CI 4.3–8.3) and 9.9% at 24 months (95% CI 7.3–12.4). Among other findings, in patients without a history of substance use disorder and patients whose sentences did not include special court supervision, recidivism was significantly higher in those with a shorter treatment duration.

**Conclusion:**

Using the entirety of a suitable, contemporary, prospectively enrolled cohort of mentally ill offenders, we were able to estimate—with greater accuracy than previous studies—the average duration of Swedish forensic psychiatric care and rate of subsequent criminal recidivism.

## 1. Introduction

Criminal offenders who are deemed to suffer from a severe mental disorder are subject to exemption under the law in most countries, albeit the corresponding definition of mental disorder and specifics of legal treatment vary (1–6). The Swedish judicial system is relatively unique in this respect, as practically all offenders are considered to be accountable for their crimes regardless of their mental status. The main difference lies in the sanction choice, whereby offenders with severe mental disorder are typically sentenced to compulsory treatment in the form of forensic psychiatric care. The Forensic Mental Care Act (7) first came into effect in 1991, followed by changes in the Swedish Criminal Code in 2008 (8). In contrast to a typical prison sentence, the duration of confinement within forensic psychiatric care is not determined at the time of sentencing; if the perpetrator of a serious (for example, violent) offense is thought to pose a risk of relapse in serious crime—as the majority of offenders are thought to do—the criminal court, without regard to the degree of illness, will impose a provision requiring special court supervision, meaning that the decision when to terminate care lies with the supervisory court and is heavily based on regular assessments of the individual’s risk of criminal recidivism. The main purpose of forensic psychiatric care is thus not only to stabilize patients’ mental status and social circumstances but also to reduce their risk of reoffending. The extent to which forensic psychiatric patients’ length of stay is, or should be, influenced by the severity of their index crime has over the years been heavily debated (9, 10); however, credible estimates of average treatment durations are still lacking in the literature. Duration of forensic psychiatric care in Sweden has thus far been presented statistically as mean time to discharge among the subset of patients who have in fact attained that milestone (11, 12); this manner of delimiting the dataset has inevitably excluded individuals with longer, not yet terminated care, thereby underestimating average treatment periods.

Two recent studies performed using a Swedish dataset have investigated time estimates for and rates of reconviction after termination of forensic psychiatric treatment, as well as risk factors associated with reconviction, including length of stay, age, and substance misuse (13, 14). However, it turns out that these studies have both ignored errors in the registry from which the dataset was derived and failed to exclude individuals who were not included in the dataset prospectively; thus, further studies—in which these errors are amended and the cohort is more stringently defined—are needed. Moreover, the previously analyzed dataset spanned two eras—each with its historically specific practices regarding sentencing, treatment regimens, and risk assessment—affecting the generalizability of calculated risk estimates and times to relapse.

A common aim for the treatment of mentally ill offenders in most countries is prevention of relapse into crime, but the particular framework for forensic psychiatric care, such as the duration of treatment, can differ quite considerably; the same is true for the subsequent recidivism rate (15–23). It is therefore of great importance to investigate how distinctive factors regarding forensic psychiatric care and offenders influence the risk of recidivism. In this study—using the entirety of a single, contemporary, prospectively enrolled cohort of mentally ill offenders—we present new estimates of the average duration of Swedish forensic psychiatric care. We first investigate the influence of important criminologico-demographic variables on treatment duration, and then, after subgrouping offenders according to these variables, explore the impact of treatment duration on criminal recidivism after discharge from care.

## 2. Materials and methods

### 2.1. Setting

We conducted a retrospective cohort study of patients registered in the Swedish National Forensic Psychiatric Register (SNFPR) who, according to the Swedish National Council for Crime Prevention’s crime registry, were sentenced to forensic psychiatric care between January 1, 2009, and December 31, 2019.

The SNFPR was established in 2008 and in 2019 covered 96% of forensic psychiatric units and 86% of all patients under forensic psychiatric care in Sweden (24). The enrollment occurs on voluntary basis with the possibility of at any time opting out. It provides a wide range of clinical and sociodemographic information at the individual level and covers the whole course of forensic psychiatric care, including transition from inpatient to outpatient care, until the moment the patient is discharged.

We used patients’ unique Swedish personal identity numbers to link the SNFPR with the Swedish National Council for Crime Prevention’s crime registry (covering all convictions in Sweden beginning in 1973) to link and obtain individual data on convictions, including the type of offense and dates of crime and sentencing. Then, the linked datasets were supplemented with information on patients who died during the study period according to the Swedish Cause of Death Register. Patients without Swedish personal identity numbers were excluded, as such numbers were necessary for the linkage of registries.

### 2.2. Study sample and relevant variables

**Figure 1:**
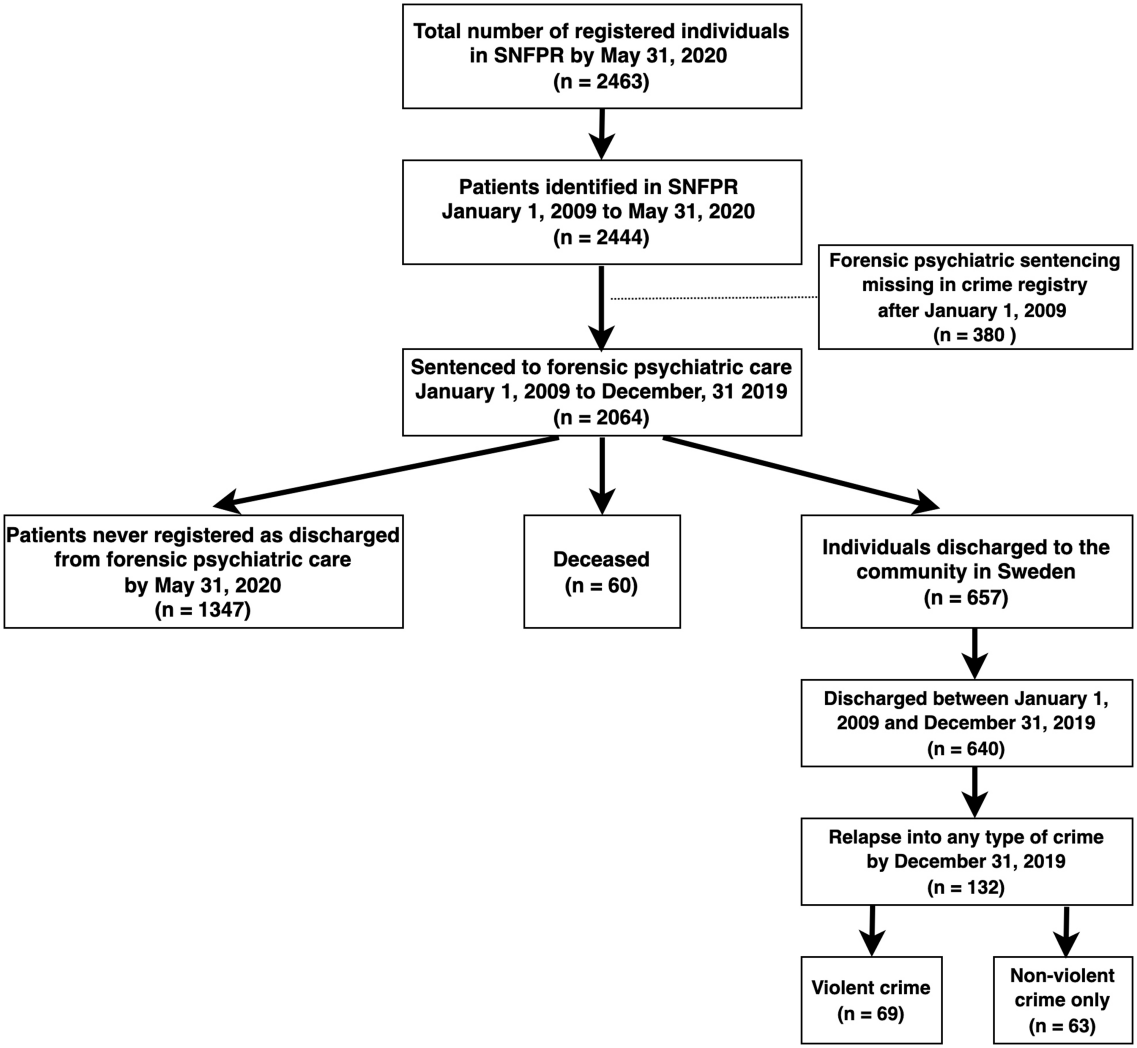
Flowchart describing the present cohort. SNFPR = Swedish National Forensic Psychiatric Register.

The SNFPR provided information on 2,444 individuals receiving forensic psychiatric care at any point between January 1, 2009, and May 31, 2020. A total of 380 of these individuals were excluded from the analysis, because a corresponding sentence after January 1, 2009, was not present in the crime registry (Figure 1).

The analysis of treatment duration included only individuals sentenced from January 1, 2009, to December 31, 2019. The variables that *a priori* were hypothesized to potentially influence treatment duration were sex, type of offense, and type of sanction (with or without special court supervision), as extracted from the crime registry; as well as diagnosis and history of substance use disorder according to data from the SNFPR. The type of offense was divided into lethal violence (murder, voluntary manslaughter, and infanticide), non-lethal violence (attempted lethal violence; assault; involuntary manslaughter; crimes against liberty and peace, such as molestation, unlawful coercion, or unlawful threat; sexual crimes, such as rape and sexual molestation, including crimes against minors; robbery; arson; violence or threat against a public official; and violent resistance) and non-violent crime (remaining offenses, such as theft and drug-related criminality). Attempted lethal violence was included in the non-lethal violence group, as it did not in fact result in death. Involuntary manslaughter was included in the same group, based on the absence of intent of harm, a factor that, in the penal system, is associated with a considerably more lenient sentence. The diagnosis was coded as “psychosis” if an ICD-10 code (25) designating a psychotic disorder (F20–F29) was registered at least once during the treatment period, and as “non-psychosis” in remaining cases. The analysis was also reperformed using an alternative set of levels: “psychosis” for psychotic disorders according to ICD-10 but with exclusion of acute and transient psychosis (F23); “developmental and personality disorders” for intellectual disabilities (F70–F79), pervasive developmental disorders (i.e., forms of autism; F84), attention-deficit hyperactivity disorder (F90), and personality disorders (F60, F61.9); and “other” for the remaining diagnoses, including affective disorders. The overall duration of forensic psychiatric care was calculated using the date of sentencing according to the crime registry and the date of final discharge from the treatment according to the SNFPR.

In the analysis of subsequent criminal reoffending, we focused on patients who were both sentenced and discharged during the period from January 1, 2009, to December 31, 2019, as the data on recidivism were not available for the period after the latter date. We used the computed duration of treatment in combination with the age listed in the crime registry at the time of sentencing in order to calculate the age at discharge. The discharged patients were divided into groups according to sex, type of original offense, type of sanction, diagnosis, and history of substance use disorder. The main exposure of interest was duration of previous forensic psychiatric care; the outcomes of interest were relapse in crime generally, and violent offending specifically. Information on reoffending in the form of date and type of crime was obtained from the crime registry, and the date of death from the Swedish Cause of Death Register.

### 2.3. Statistical analysis

The analysis was separated into two steps: determination of duration of treatment and evaluation of subsequent recidivism. We calculated descriptive statistics and used the Kaplan–Meier estimator to calculate median duration of treatment and visualized time within forensic psychiatric care for the entire dataset, as well as for levels of specific variables. The log-rank test was used to assess statistical differences between the levels. The analysis of recidivism focused on two outcomes, according to the type of crime committed after discharge from forensic psychiatric care: the earliest crime, irrespective of the type of offense, and the earliest violent crime. We used the Kaplan–Meier estimator to evaluate and visualize the cumulative incidence of recidivism both overall and then stratified for sex, original offense, type of sentence, diagnosis, and history of substance use disorder, and dichotomized within each stratum based on median treatment duration. We assessed statistical differences between treatment-duration subgroups using the log-rank test. Uncorrected two-sided probability values less than 0.05 were considered statistically significant. All calculations and analyses were conducted using R version 4.0.5.

### 2.4. Ethics approval

Ethical approval for this study was obtained from the Swedish Ethical Review Authority (case number 2019–04048). All data were pseudonymized and as such could no longer be attributed to a specific person.

## 3. Results

### 3.1. Duration of forensic psychiatric care

#### 3.1.1. Cohort description

In the SNFPR were registered 2064 individuals who were sentenced to forensic psychiatric care between January 2009 and December 2019 according to the crime registry. Twenty-one patients were discharged and then sentenced again to forensic psychiatric treatment during the follow-up period; as such observations cannot be considered independent, for each patient, only the first treatment period was included in our analysis.

Of the 2064 individuals in total, 1,697 (82.2%) were men. Sentences of forensic psychiatric care were most commonly based on non-lethal violent crimes (1770 cases; 85.8%), followed by non-violent crimes (225 cases; 10.9%) and lethal violent crimes (69 individuals; 3.3%). In 1581 cases (76.6%), the sentence of forensic psychiatric care included a provision requiring special court supervision, while 1,530 individuals (74.1%) were diagnosed with psychosis during the treatment period, and 1,267 (62.9%) had a history of substance use disorder (Table 1).

**Table 1:** Cohort characteristics—duration of forensic psychiatric care.

Forensic psychiatric care January 2009–May 2020		All patients (*n* = 2064)	Discharged (*n* = 657)
Sex			
	Male	1,697 (82.2%)	522 (79.5%)
	Female	367 (17.8%)	135 (20.5%)
Diagnosis			
	Psychosis	1,530 (74.1%)	452 (68.8%)
	Non-psychosis	534 (25.9%)	205 (31.2%)
Alternative levels	Psychosis (excluding acute and transient psychosis)	1,469 (71.2%)	428 (65.1%)
	Developmental and personality disorders	411 (19.9%)	133 (20.2%)
	Other (including affective disorders)	184 (8.9%)	96 (14.6%)
Original offense			
	Lethal violence	69 (3.3%)	12 (1.8%)
	Non-lethal violence	1770 (85.8%)	542 (82.5%)
	Non-violence	225 (10.9%)	103 (15.7%)
Special court supervision			
	Yes	1,581 (76.6%)	409 (62.3%)
	No	483 (23.4%)	248 (37.7%)
History of substance use disorder*			
	Yes	1,267 (62.9%)	336 (52.9%)
	No	746 (37.1%)	299 (47.1%)
		*n* = 2013	*n* = 635

*51 missing values.

#### 3.1.2. Treatment duration

**Figure 2:**
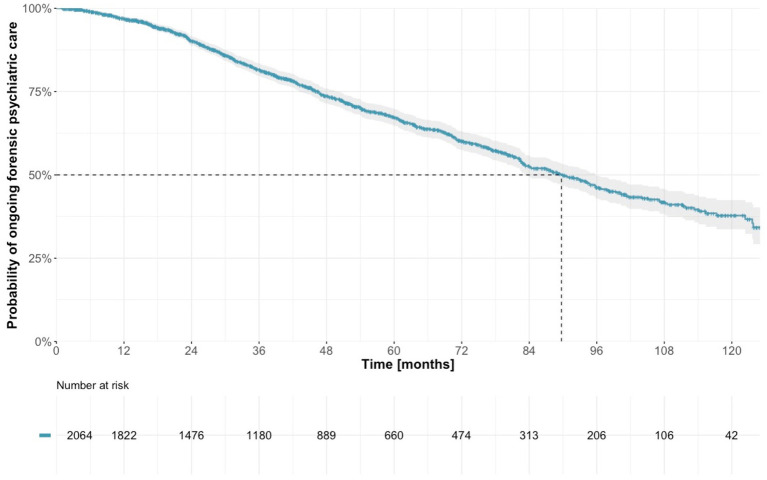
Estimated time from sentence to discharge from forensic psychiatric care.

There were 657 patients registered as discharged from forensic psychiatric treatment between January 2009 and May 2020. The median duration of treatment, calculated using the Kaplan–Meier estimator, was 89.7 months (95% CI 83.2–95.8; Table 2; Figure 2).

**Table 2:** Estimated percentages of discharged patients over time, with 95% confidence intervals (CI).

Treatment duration (months)	Probability of being discharged (%)	95% CI
12	3.2	2.5–4.0
24	9.8	8.4–11.2
36	18.4	16.5–20.2
48	26.3	24.1–28.5
60	32.7	30.2–35.2
72	39.8	36.9–42.5
84	47.5	44.3–50.5
96	53.8	50.2–57.1
108	58.1	54.2–61.7
120	62.2	57.6–66.3

**Figure 3:**
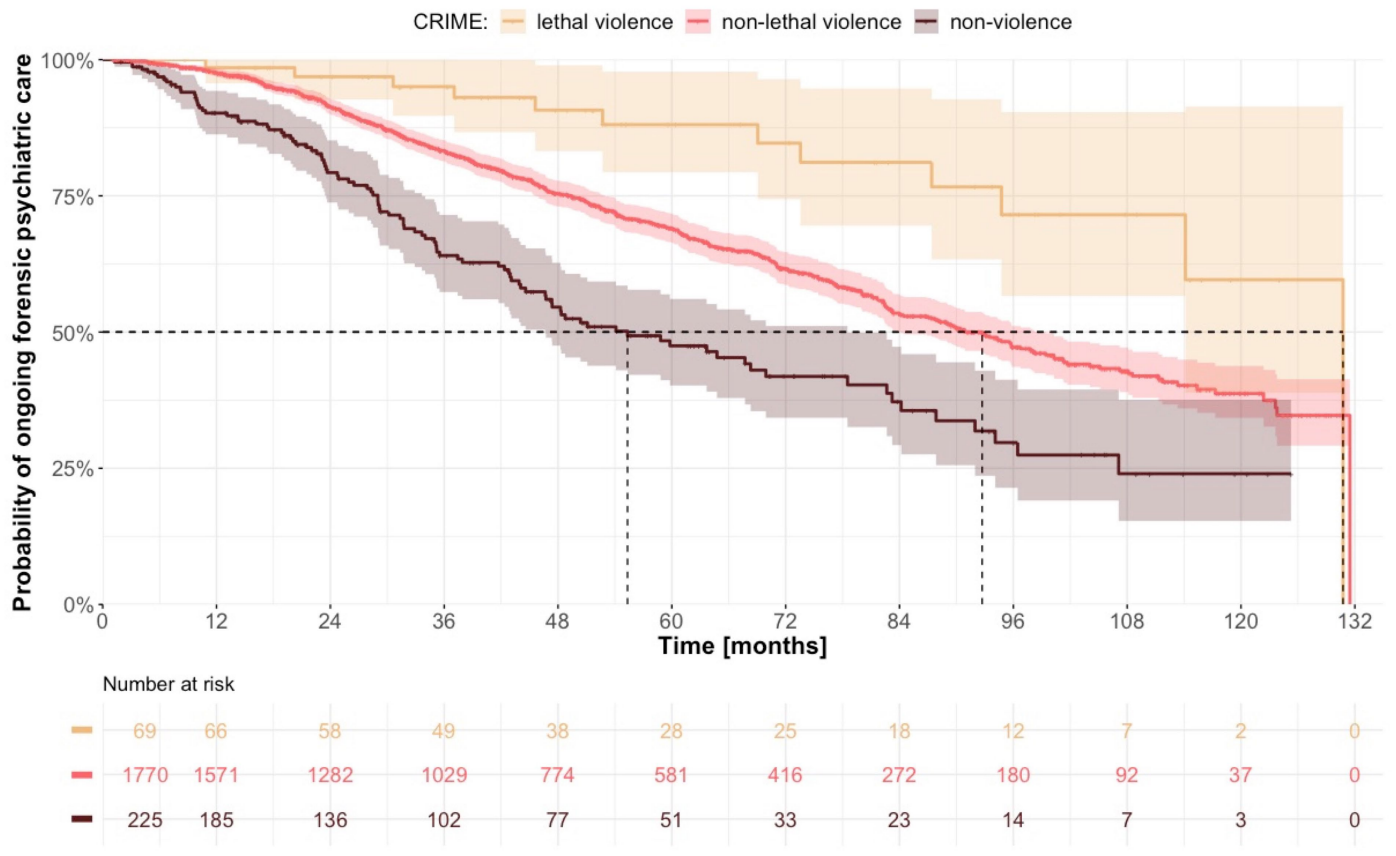
Estimated time from sentence to discharge from forensic psychiatric care with regard to original crime.

**Figure 4:**
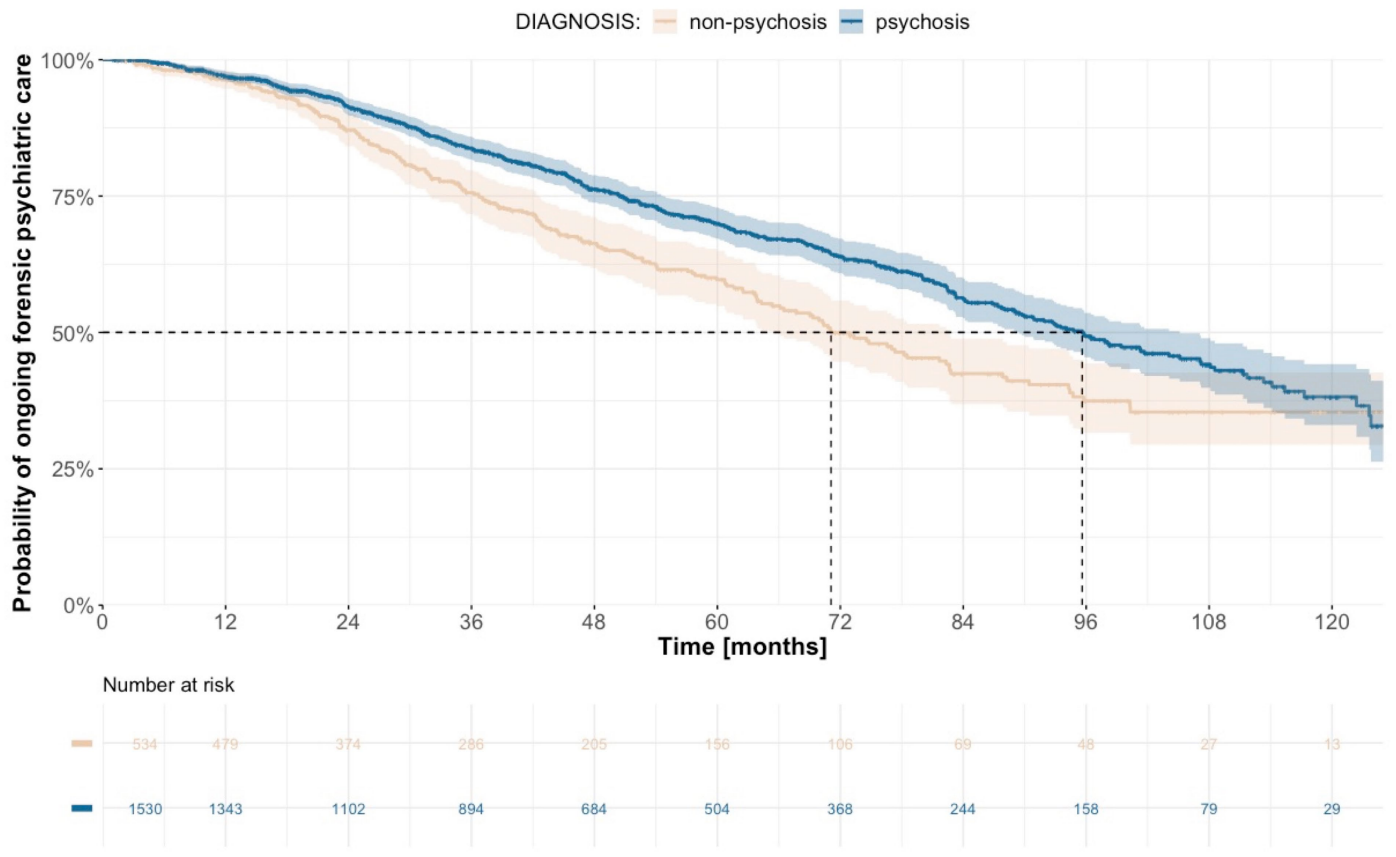
Estimated time from sentence to discharge from forensic psychiatric care with regard to diagnosis.

Offenders who were sentenced on the basis of a lethal violent crime had the longest median duration of treatment, at 130.8 months (95% CI 114.1-not calculable), compared to 92.7 months (95% CI 84.0–99.4) for non-lethal violent crime and 55.3 months (95% CI 46.7–78.5) for non-violent crime (Figure 3). The median duration of treatment was higher for individuals sentenced to forensic psychiatric care with special court supervision: 104.1 months (95% CI 95.7–115.4) vs. 44.6 months (95% CI 38.1–54.5) for sentences without special court supervision (Supplementary Figure 1). When the dataset was analyzed according to diagnosis, we noted longer median duration of treatment for individuals who were diagnosed with psychosis (95.6 months; 95% CI 89.5–105.1) compared to individuals without such a diagnosis (71.1 months; 95% CI 65.3–82.7; Figure 4). With the alternative division of diagnoses, the length of forensic psychiatric care was relatively similar in the psychosis group (95.8 months; 95% CI 89.5–107.1) and the developmental and personality disorders group (90.4 months; 95% CI 77.3-not calculable), but much shorter among patients with other diagnoses (42.5 months; 95% CI 33.4–54.5; Supplementary Figure 13). History of substance use disorder was also associated with longer median treatment period: 99.4 months (95% CI 92.0–114.1) vs. 75.5 months (95% CI 68.6–84.4; Supplementary Figure 2). The median duration of forensic psychiatric care was 80.7 months (95% CI 75.9–93.5) in women and 94.1 months (95% CI 84.4–100.3) in men, a difference that was not significant (Supplementary Figure 3, Table 3).

**Table 3:** Estimated median treatment duration with regard to criminologico-demographic variables, with probability (*p*) values comparing levels of each variable calculated using log-rank test.

Treatment duration		Median (months)	95% CI	*p* value
Sex				0.1
	Male	94.1	84.4–100.3	
	Female	80.7	75.9–93.5	
Diagnosis				<0.0001
	Psychosis	95.6	89.5–105.1	
	Non-psychosis	71.1	65.3–82.7	
Alternative levels	Psychosis (excluding acute and transient psychosis)	95.8	89.7–107.1	<0.0001*
	Developmental and personality disorders	90.4	77.3 - NC	
	Other (including affective disorders)	42.5	33.4–54.5	
Original offense				<0.0001**
	Lethal violence	130.8	114.1 – NC	
	Non-lethal violence	92.7	84.0–99.4	
	Non-violence	55.3	46.7–78.5	
Special court supervision				<0.0001
	Yes	104.1	95.7–115.4	
	No	44.6	38.1–54.5	
History of substance use disorder***				<0.0001
	Yes	99.4	92.0–114.1	
	No	75.5	68.6–84.4	

NC = not calculable.

* Psychosis vs. developmental and personality disorders and other.

** Violent crime (lethal violence and non-lethal violence) vs. non-violent crime.

*** 51 missing values.

### 3.2. Criminal recidivism after discharge from treatment

#### 3.2.1. Cohort description

Between January 1, 2009, and December 31, 2019, 640 patients were discharged from forensic psychiatric care, 508 (79.4%) of whom were men. Sentences of forensic psychiatric care were most commonly based on non-lethal violent crimes (529 cases; 82.7%), followed by non-violent crimes (101 cases; 15.8%) and lethal violent crimes (10 cases; 1.6%). For 396 individuals (61.9%), the sentence of forensic psychiatric care included a provision requiring special court supervision. In total, 438 individuals (68.4%) were diagnosed with psychosis during the treatment period, and 325 (52.5%) had a history of substance use disorder. The median duration of forensic psychiatric care was 37.8 months (with values ranging from 1.2 months to 123.8 months), and the mean age at the time of discharge was 42.3 years (SD 13.8; Table 4).

**Table 4:** Cohort characteristics—criminal recidivism (all crimes).

Criminal recidivism after discharge from treatment January 2009–December 2019		All discharged (*n* = 640)	Reoffended (*n* = 132)	Not reoffended (*n* = 508)
Sex				
	Male	508 (79.4%)	107 (81.1%)	401 (78.9%)
	Female	132 (20.6%)	25 (18.9%)	107 (21.1%)
Mean age at discharge, in years (SD)				
		42.3 (13.8)	37.6 (12.1)	43.6 (14.0)
Median treatment duration, in months (minimum - maximum)				
		37.8 (1.2–123.8)	27.4 (1.2–107.2)	41.0 (2.9–123.8)
Diagnosis				
	Psychosis	438 (68.4%)	92 (69.7%)	346 (68.1%)
	Non-psychosis	202 (31.6%)	40 (30.3%)	162 (31.9%)
Alternative levels	Psychosis (excluding acute and transient psychosis)	415 (64.8%)	88 (66.7%)	327 (64.4%)
	Developmental and personality disorders	130 (20.3%)	32 (24.2%)	98 (19.3%)
	Other (including affective disorders)	95 (14.8%)	12 (9.1%)	83 (16.3%)
Original offense				
	Lethal violence	10 (1.6%)	0 (0.0%)	10 (2.0%)
	Non-lethal violence	529 (82.7%)	102 (77.3%)	427 (84.1%)
	Non-violence	101 (15.8%)	30 (22.7%)	71 (14.0%)
Special court supervision				
	Yes	396 (61.9%)	61 (46.2%)	335 (65.9%)
	No	244 (38.1%)	71 (53.8%)	173 (34.1%)
History of substance use disorder*				
	Yes	325 (52.5%)	93 (72.1%)	232 (47.3%)
	No	294 (47.5%)	36 (27.9%)	258 (52.7%)
		*n* = 619	*n* = 129	*n* = 490

*21 missing values.

#### 3.2.2. Criminal recidivism—Any type of crime

**Figure 5:**
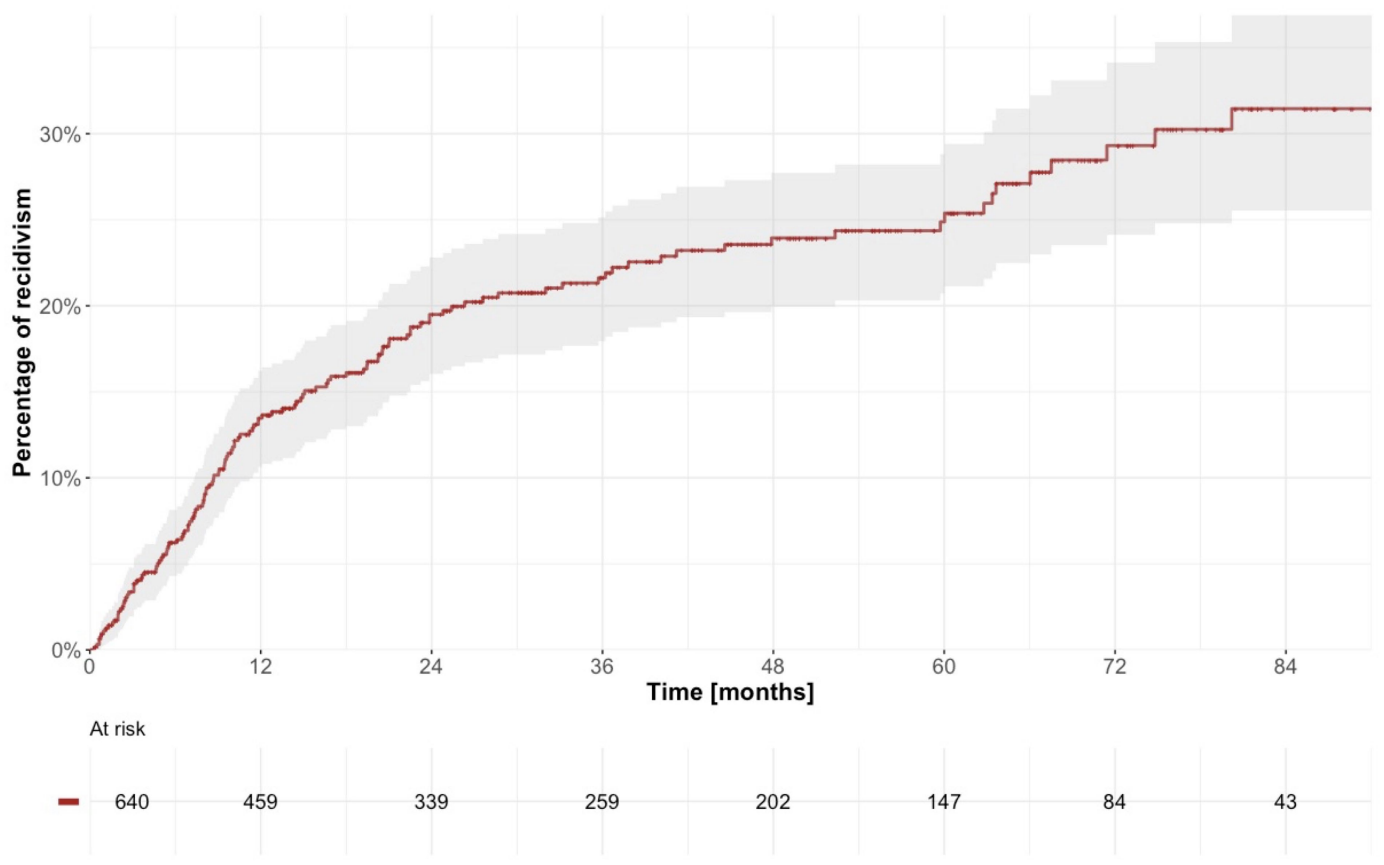
Estimated time to reoffending after discharge from forensic psychiatric care.

**Figure 6:**
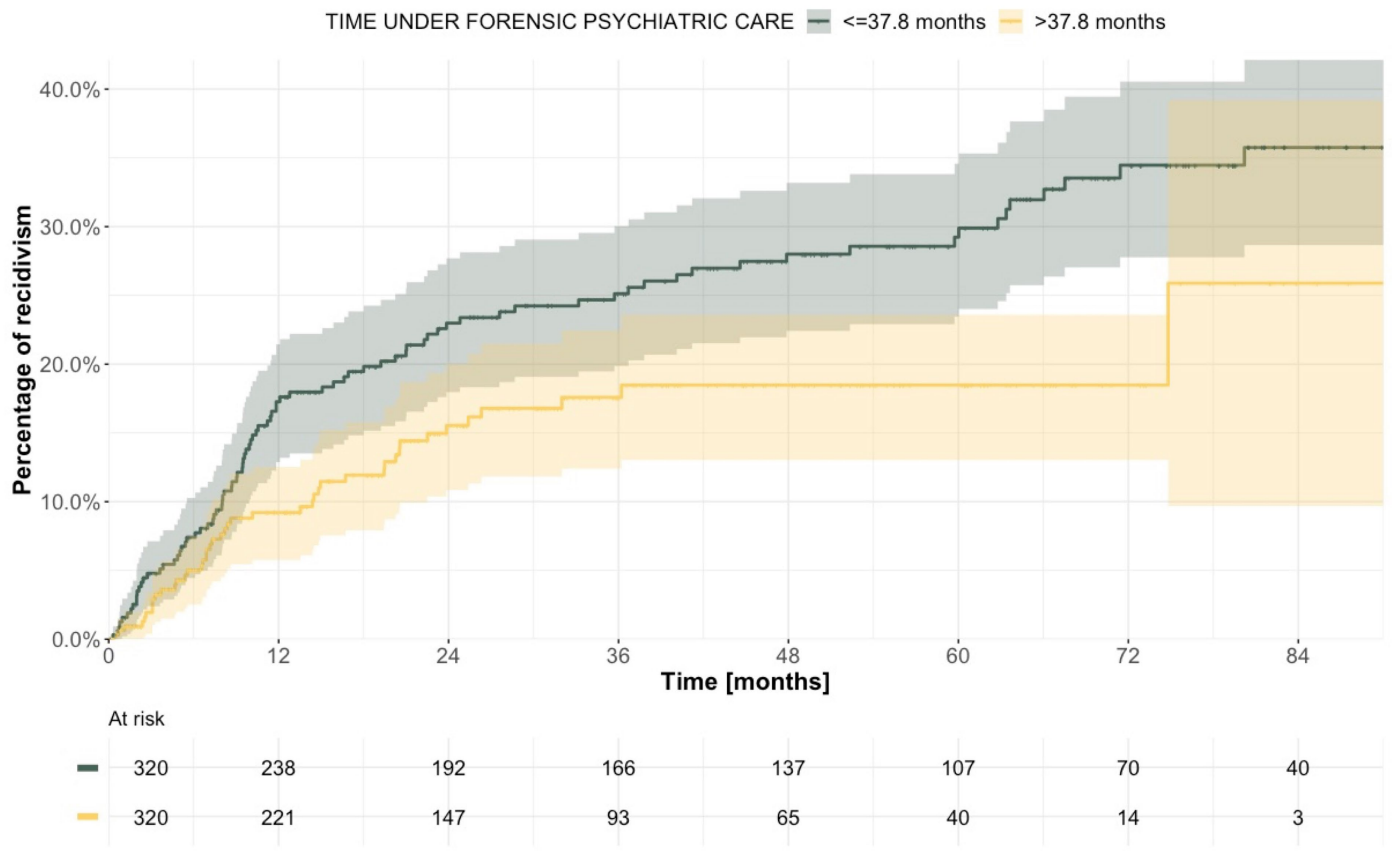
Estimated time to reoffending after discharge from forensic psychiatric care after dichotomization according to treatment duration.

In total, 132 individuals (20.6%) relapsed in crime after discharge from forensic psychiatric care. The first offense after discharge involved non-lethal violence in 45 individuals (34.1%), whereas the remaining 87 individuals (65.9%) relapsed in a non-violent crime; no lethal violence was observed. In total, 107 (81.1%) of the reoffenders were men. Median follow-up time for all discharged patients was 26.3 months (with values ranging from 0.0 to 117.2), and the recidivism rate was 7,132 instances of reoffending per 100,000 person-years (95% CI 5967–8,458). The cumulative incidence of recidivism was estimated to 13.5% at 12 months (95% CI 10.6–16.2), 19.5% at 24 months (95% CI 16.0–22.8) and 24.9% at 60 months (95% CI 20.7–28.8; Table 5; Figure 5). When the whole cohort of discharged patients was dichotomized around the median according to treatment duration, we observed a statistically significantly higher risk of criminal recidivism in patients with shorter treatment duration (Figure 6).

**Table 5:** Estimated percentages of criminal recidivism over time, with 95% confidence intervals (CI).

Months after discharge	Recidivism, any crime %	95% CI	Recidivism, violent crime %	95% CI
12	13.5	10.6–16.2	6.3	4.3–8.3
24	19.5	16.0–22.8	9.9	7.3–12.4
36	21.6	17.9–25.1	11.6	8.7–14.4
48	23.9	20.0–27.7	13.2	10.0–16.3
60	24.9	20.7–28.8	13.6	10.3–16.7
72	29.3	24.1–34.1	15.3	11.5–18.9
84	31.5	25.5–36.9	16.4	12.1–20.5

**Figure 7:**
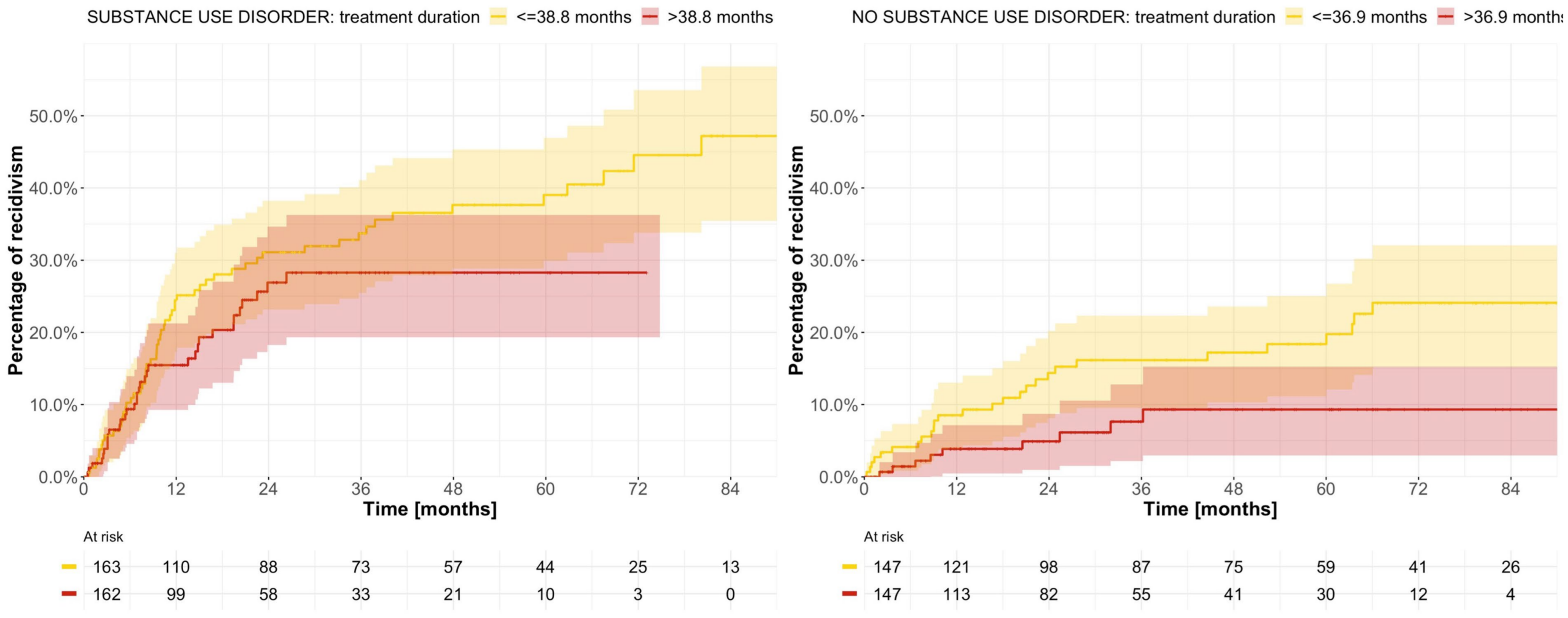
Estimated time to reoffending after discharge from forensic psychiatric care after stratification by history of substance use disorder and dichotomization according to treatment duration.

Median duration of previous forensic psychiatric care was 37.4 months (with values ranging from 2.9 to 123.8) for men, and 41.6 months (with values ranging from 1.2 to 105.1) for women. The difference in recidivism in relation to duration of care (shorter than or equal to vs. longer than median) was statistically significant for men but not for women (Supplementary Figure 4). When stratified according to type of crime, the median treatment duration was 39.4 months (with values ranging from 2.9 to 123.8) for those originally sentenced to forensic psychiatric care on the basis of a lethal or non-lethal violent crime, and 29.2 months (with values ranging from 1.2 to 107.1) for those sentenced on the basis of a non-violent crime (Supplementary Figure 5). Median duration of forensic psychiatric care was 46.7 months (with values ranging from 4.8 to 123.8) in patients whose sentence included special court supervision, yet much shorter in patients without such supervision, at 25.1 months (with values ranging from 1.2 to 101.3). In the latter group, we observed a significant difference in recidivism when it was dichotomized based on duration of care, whereby those with shorter treatment relapsed in crime more often (Supplementary Figure 6). After stratification according to diagnosis, median treatment duration was 39.4 months (with values ranging from 3.0 to 123.8) for patients with psychosis and 34.8 months (with values ranging from 1.2 to 100.3) for patients with other diagnoses (Supplementary Figure 7), with statistically significant differences in criminal recidivism according to the length of treatment in patients with psychosis. When the alternative grouping was used, the statistical significance could no longer be seen for the psychosis group but was instead observed among patients with developmental and personality disorders, with mean treatment duration 41.2 months (with values ranging from 2.9 to 100.3; Supplementary Figure 14). Patients with a history of substance use disorder had a median treatment duration of 38.8 months (with values ranging from 2.9 to 123.8), whereas in patients without such a history, the median duration of forensic psychiatric care was 36.9 months (with values ranging from 1.2 to 113.3). In patients without a history of substance use disorder, criminal recidivism was statistically significantly higher in the group with shorter treatment duration (Table 6; Figure 7).

**Table 6:** Criminal recidivism stratified for relevant variables and dichotomized within each stratum based on median treatment duration, with probability (*p*) values comparing effect of treatment duration using log-rank test.

Criminal recidivism according to treatment duration		Median treatment duration (months)	Dichotomization according to treatment duration	Reoffended (*n*)	*p* value
All *n* = 640		37.8	shorter/equal to median	88	0.009
			longer than median	44	
Sex	Male *n* = 107	37.4	shorter/equal to median	69	0.048
			longer than median	38	
	Female *n* = 25	41.6	shorter/equal to median	18	0.2
			longer than median	7	
Diagnosis	Psychosis *n* = 92	39.4	shorter/equal to median	62	0.048
			longer than median	30	
	Non-psychosis *n* = 40	34.8	shorter/equal to median	26	0.1
			longer than median	14	
Alternative levels	Psychosis (excluding acute and transient psychosis) *n* = 88	41.7	shorter/equal to median	50	0.1
			longer than median	30	
	Developmental and personality disorders *n* = 32	41.2	shorter/equal to median	23	0.031
			longer than median	9	
	Other (including affective disorders) *n* = 12	28.3	shorter/equal to median	9	0.1
			longer than median	3	
Original offense	Violent crime *n* = 102	39.4	shorter/equal to median	66	0.1
			longer than median	36	
	Non-violent crime *n* = 30	29.2	shorter/equal to median	18	0.5
			longer than median	12	
Special court supervision	Yes *n* = 61	46.7	shorter/equal to median	32	0.3
			longer than median	29	
	No *n* = 71	25.1	shorter/equal to median	48	0.02
			longer than median	23	
History of substance use disorder*	Yes *n* = 93	38.8	shorter/equal to median	58	0.3
			longer than median	35	
	No *n* = 36	36.9	shorter/equal to median	27	0.02
			longer than median	9	

* 21 missing values.

#### 3.2.3. Criminal recidivism—Violent crime

Of the 640 discharged individuals, 69 (10.8%) committed at least one violent crime after discharge from forensic psychiatric care, 55 (79.7%) of whom were men (Table 7). Median follow-up time was 32.5 months (with values ranging from 0.0 to 117.2), and the offending rate was 3,294 per 100,000 person years (95% CI 2563–4,169). The estimated cumulative incidence of violent crime was 6.3% at 12 months (95% CI 4.3–8.3), 9.9% at 24 months (95% CI 7.3–12.4) and 13.6% at 60 months (95% CI 10.3–16.7; Table 5; Supplementary Figure 8).

**Table 7:** Cohort characteristics—criminal recidivism (violent crimes).

Violent crime after discharge from treatment January 2009–December 2019		All discharged (*n* = 640)	Reoffended with violent crime (*n* = 69)	No violent crime (*n* = 571)
Sex				
	Male	508 (79.4%)	55 (79.7%)	453 (79.3%)
	Female	132 (20.6%)	14 (20.3%)	118 (20.7%)
Mean age at discharge, in years (SD)				
		42.3 (13.8)	36.4 (12.2)	43.1 (13.9)
Median treatment duration, in months (minimum - maximum)				
		37.8 (1.2–123.8)	23.9 (3.0–107.2)	39.1 (1.2–123.8)
Diagnosis				
	Psychosis	438 (68.4%)	41 (59.4%)	397 (69.5%)
	Non-psychosis	202 (31.6%)	28 (40.6%)	174 (30.5%)
Alternative levels	Psychosis (excluding acute and transient psychosis)	415 (64.8%)	38 (55.1%)	377 (66%)
	Developmental and personality disorders	130 (20.3%)	25 (36.2%)	105 (18.4%)
	Other (including affective disorders)	95 (14.8%)	6 (8.7%)	89 (15.6%)
Original offense				
	Lethal violence	10 (1.6%)	0 (0.0%)	10 (1.8%)
	Non-lethal violence	529 (82.7%)	60 (87.0%)	469 (82.1%)
	Non-violence	101 (15.8%)	9 (13.0%)	92 (16.1%)
Special court supervision				
	Yes	396 (61.9%)	36 (52.2%)	360 (63.0%)
	No	244 (38.1%)	33 (47.8%)	211 (37.0%)
History of substance use disorder*				
	Yes	325 (52.5%)	49 (73.1%)	276 (50.0%)
	No	294 (47.5%)	18 (26.9%)	276 (50.0%)
		*n* = 619	*n* = 67	*n* = 552

*21 missing values.

**Figure 8:**
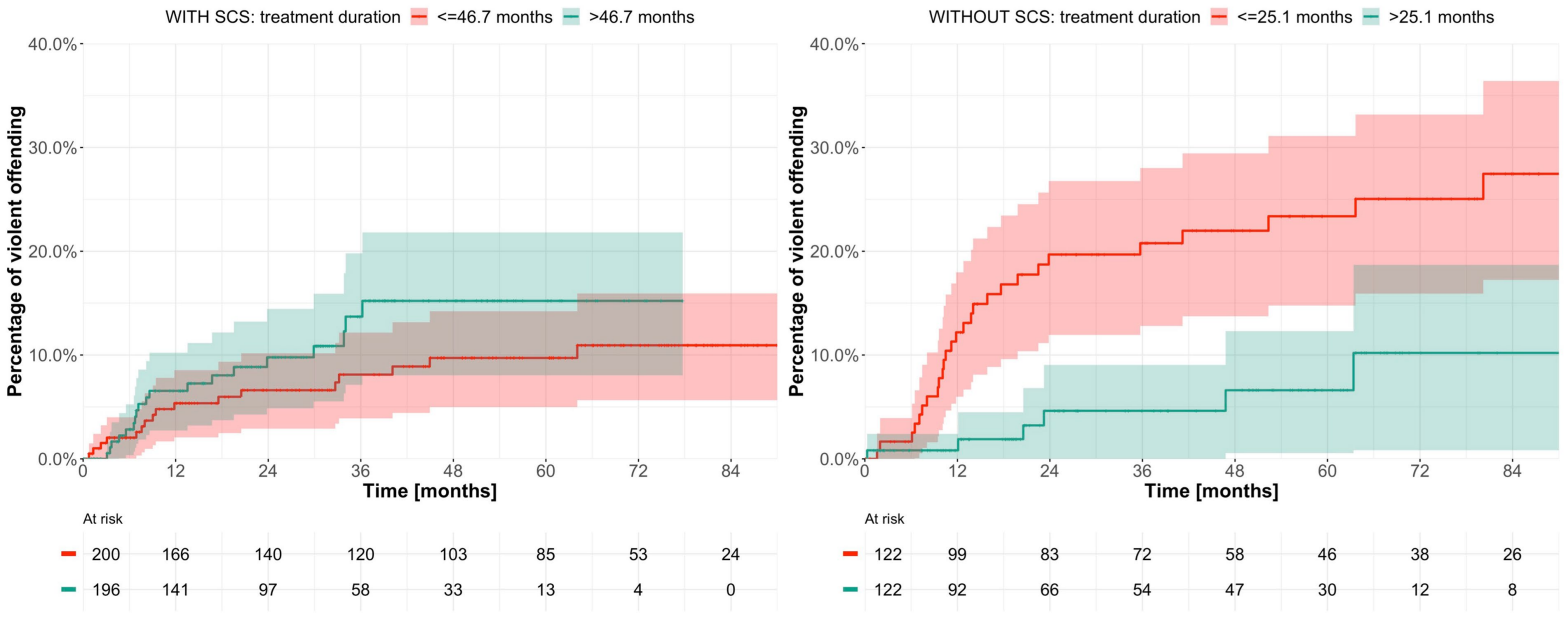
Estimated time to violent offending after discharge from forensic psychiatric care after stratification by sentence type and dichotomization according to treatment duration. SCS = special court supervision.

When the cohort was alternately stratified according to sex, original crime, type of sentence, presence of psychosis, and history of substance use disorder, and then dichotomized according to treatment duration, no significant differences in violent offending were observed, with one exception: among patients whose sentence did not include special court supervision, treatment duration shorter than or equal to the median (25.1 months) was associated with a higher probability of committing a violent crime (probability value 0.001; Figure 8; Supplementary Figures 9–12).

## 4. Discussion

### 4.1. Key findings in context

In the current study, using a large sample of mentally ill offenders enrolled prospectively in a nationwide registry, we present intuitive and useful estimates of average duration of forensic psychiatric care in Sweden. The median duration of forensic psychiatric care in the entire sample was estimated to approximately 7.5 years. Offenders who committed violent crimes, suffered from psychosis, or had a history of substance use disorder, and in offenders whose sentences included special court supervision had a longer treatment duration than offenders in whom these characteristics were absent. The debate in Sweden regarding sentences of forensic psychiatric care has in large part revolved around the proportionality of such sanctions in a larger, juridical, and ethical context. More specifically, the questions under debate have been the following: Is the duration of a sentence of forensic psychiatric care, on average, shorter, or longer than a prison sentence meted out for the same offense? (9, 11) And, is the treatment duration—from a medical point of view—needlessly extended in an effort to attain proportionality between types of sanctions? These questions remain unanswered, but the present study represents a necessary first step toward addressing these issues. Indeed, in the near future, we plan to publish results of an analysis of offenders who had undergone forensic psychiatric evaluation, with a view to—after matching for sex, age, and history of substance use disorder—comparing respective sanction durations for similar offenses. Further, in the current study, shorter treatment duration was, among men, patients with psychosis, patients without a history of substance use disorder and patients whose sentences did not include special court supervision, significantly associated with greater probability of criminal recidivism after discharge; however, the performed univariable analyses were unable to explore the possible interdependence of the variables for which the cohort was alternately stratified.

### 4.2. Strengths and limitations

One of the principal strengths of our study is its statistical design, whereby survival function was used to calculate median treatment duration. Previous studies based their assessments on patients already discharged from forensic psychiatric care, thus underestimating the average duration, since those with very long treatment periods were still in care and therefore excluded. Another strength of our study is its large sample size and the high coverage of the registries; as such, it can be considered as representative for the contemporary Swedish forensic psychiatric system. Indeed, prior studies focusing on this topic have failed to account for the effect of differences in legal and clinical treatment of mentally ill offenders sentenced in different historical eras (13, 14). Here, we decided to restrict our analysis to patients who were sentenced under the current legislation and enrolled in the cohort prospectively, thus making the sample more homogeneous, the results more intuitive and the conclusions more relevant to future deliberations on forensic psychiatric care. Moreover, our reanalysis of the effect of diagnosis on treatment duration and recidivism risk—after removal from the” psychosis” group of patients who in absence of chronic psychosis had been afflicted with transient psychosis—will allow comparison with results from other countries, including Finland, where according to law only individuals with chronic psychosis can be sentenced to forensic psychiatric care.

At the same, we cannot ignore the possibility that patients who declined to be enrolled in the SNFPR were more likely to possess some characteristic that affected either treatment duration, the likelihood of recidivism, or both—for example, a more general unwillingness to cooperate with others, during and after treatment. In the absence of data regarding such unwilling subjects, even cohort- and survival-based analyses will underestimate the true extent of these outcomes. Other limitations of our study are related to the fact that SNFPR relies on manual records, registered separately from each forensic psychiatric center. As a result, the accuracy of reported data can be compromised by human error. Actual dates for hospital admissions were in many cases missing or incorrect, when compared to dates of sentencing. Our choice to use the date of sentencing as representing the start of forensic psychiatric care amends this problem but could, in some cases, lead to overestimation of the total duration of the sentence.

### 4.3. Future research

Overall, criminal recidivism after discharge from forensic psychiatric care appears to be relatively low, compared to rates in offenders sentenced to prison (26). Future studies should therefore focus on exploring specific differences between these two types of sanctions and their possible effect on subsequent relapse in crime, especially (severe) violence. Also of interest is to investigate whether, in accordance with earlier findings, pharmacoadherence (27), or mere access to psychotropic medication, differentially confers protection from offending between these specific offender populations, and if such knowledge may improve the prediction accuracy of current risk assessment tools (28). Additionally, international comparisons between legal and clinical approaches to treatment and their effect on risk of relapse in crime are warranted, as criminal recidivism in mentally ill offenders appears to differ considerably between countries. Finally, a more detailed and comprehensive analysis of specific aspects of forensic psychiatric care (29, 30) and aftercare (31, 32)—which, at the same time, takes into consideration patient characteristics (33–35)—in relation to subsequent recidivism could also provide guidance for clinicians in their daily practice, as well as for policymakers engaged in deliberations on legal treatment of mentally ill offenders.

## Data availability statement

The data analyzed in this study is subject to the following licenses/restrictions: All data is subject to ethical approval and cannot be shared with third parties, by law. Requests to access these datasets should be directed to Magnus Kristiansson, magnus.kristiansson@rvn.se.

## Ethics statement

The studies involving human participants were reviewed and approved by Regional Ethics Committee in Stockholm, Sweden. Written informed consent from the participants’ legal guardian/next of kin was not required to participate in this study in accordance with the national legislation and the institutional requirements.

## Author contributions

LS, JF, and TM drafted and critically revised the paper. JF and TM conceived and designed the study. JF managed the project, including seeking ethics approval. LS conducted the analysis. All authors contributed to the article and approved the submitted version.

## Conflict of interest

The authors declare that the research was conducted in the absence of any commercial or financial relationships that could be construed as a potential conflict of interest.

## Publisher’s note

All claims expressed in this article are solely those of the authors and do not necessarily represent those of their affiliated organizations, or those of the publisher, the editors and the reviewers. Any product that may be evaluated in this article, or claim that may be made by its manufacturer, is not guaranteed or endorsed by the publisher.

## Supplementary material

The Supplementary material for this article can be found online at: https://www.frontiersin.org/articles/10.3389/fpsyt.2023.1129993/full#supplementary-material

SUPPLEMENTARY FIGURE 1 Estimated time from sentence to discharge from forensic psychiatric care with regard to sentence type.

SUPPLEMENTARY FIGURE 2 Estimated time from sentence to discharge from forensic psychiatric care with regard to history of substance use disorder.

SUPPLEMENTARY FIGURE 3 Estimated time from sentence to discharge from forensic psychiatric care with regard to sex.

SUPPLEMENTARY FIGURE 4 Estimated time to reoffending after discharge from forensic psychiatric care after stratification by sex and dichotomization according to treatment duration.

SUPPLEMENTARY FIGURE 5 Estimated time to reoffending after discharge from forensic psychiatric care after stratification by original crime and dichotomization according to treatment duration.

SUPPLEMENTARY FIGURE 6 Estimated time to reoffending after discharge from forensic psychiatric care after stratification by sentence type and dichotomization according to treatment duration. SCS = special court supervision.

SUPPLEMENTARY FIGURE 7 Estimated time to reoffending after discharge from forensic psychiatric care after stratification by diagnosis and dichotomization according to treatment duration.

SUPPLEMENTARY FIGURE 8 Estimated time to violent offending after discharge from forensic psychiatric care.

SUPPLEMENTARY FIGURE 9 Estimated time to violent offending after discharge from forensic psychiatric care after stratification by original crime and dichotomization according to treatment duration.

SUPPLEMENTARY FIGURE 10 Estimated time to violent offending after discharge from forensic psychiatric care after stratification by diagnosis and dichotomization according to treatment duration.

SUPPLEMENTARY FIGURE 11 Estimated time to violent offending after discharge from forensic psychiatric care after stratification by sex and dichotomization according to treatment duration.

SUPPLEMENTARY FIGURE 12 Estimated time to violent offending after discharge from forensic psychiatric care after stratification by history of substance use disorder and dichotomization according to treatment duration.

SUPPLEMENTARY FIGURE 13 Estimated time from sentence to discharge from forensic psychiatric care with regard to diagnosis (alternative coding). PD + NP = developmental and personality disorders.

SUPPLEMENTARY FIGURE 14 Estimated time to reoffending after discharge from forensic psychiatric care after stratification by diagnosis (alternative coding) and dichotomization according to treatment duration. PD + NP = developmental and personality disorders.

SUPPLEMENTARY FIGURE 15 Estimated time to violent offending after discharge from forensic psychiatric care after stratification by diagnosis (alternative coding) and dichotomization according to treatment duration. PD + NP = developmental and personality disorders.
