# Supplementary figures and images for: Duration of forensic psychiatric care and subsequent criminal recidivism in individuals sentenced in Sweden between 2009 and 2019
Source: Front Psychiatry. 2023 Mar 14;14:1129993. doi: 10.3389/fpsyt.2023.1129993 (PMC10053040; doi:10.3389/fpsyt.2023.1129993)

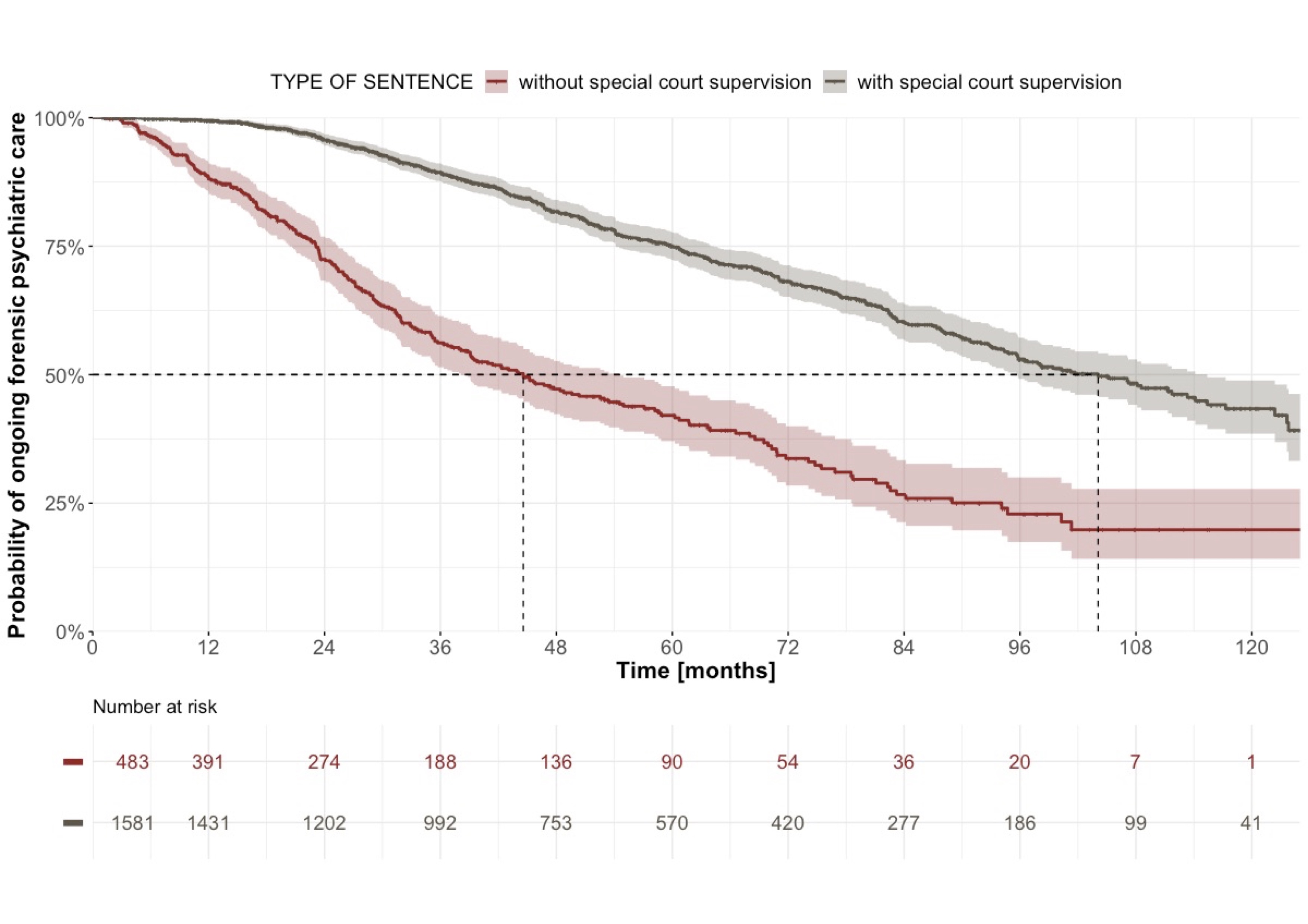

Supplement: SUPPLEMENTARY FIGURE 1 — Estimated time from sentence to discharge from forensic psychiatric care with regard to sentence type. [file Image_1.JPEG]

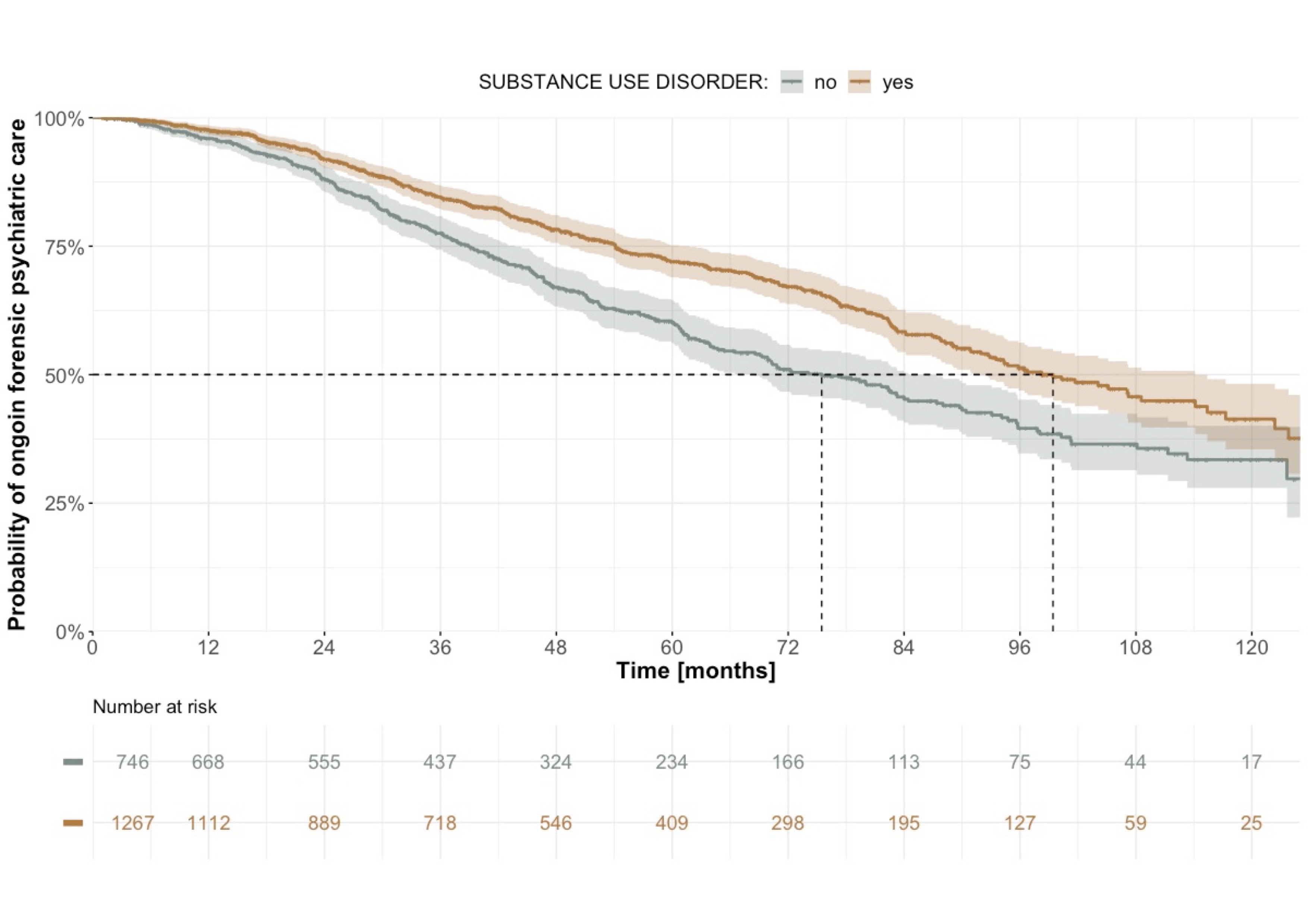

Supplement: SUPPLEMENTARY FIGURE 2 — Estimated time from sentence to discharge from forensic psychiatric care with regard to history of substance use disorder. [file Image_2.JPEG]

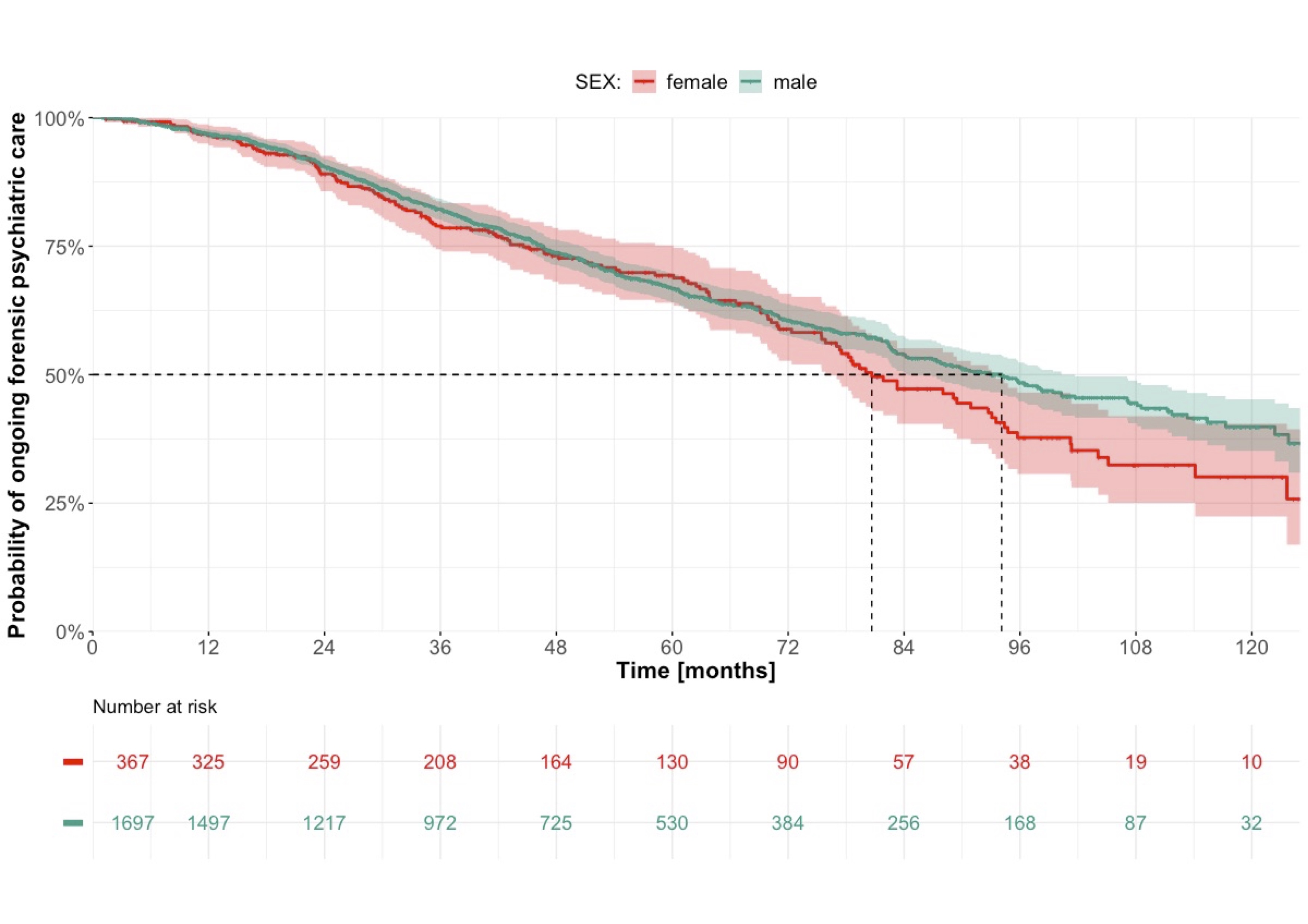

Supplement: SUPPLEMENTARY FIGURE 3 — Estimated time from sentence to discharge from forensic psychiatric care with regard to sex. [file Image_3.JPEG]

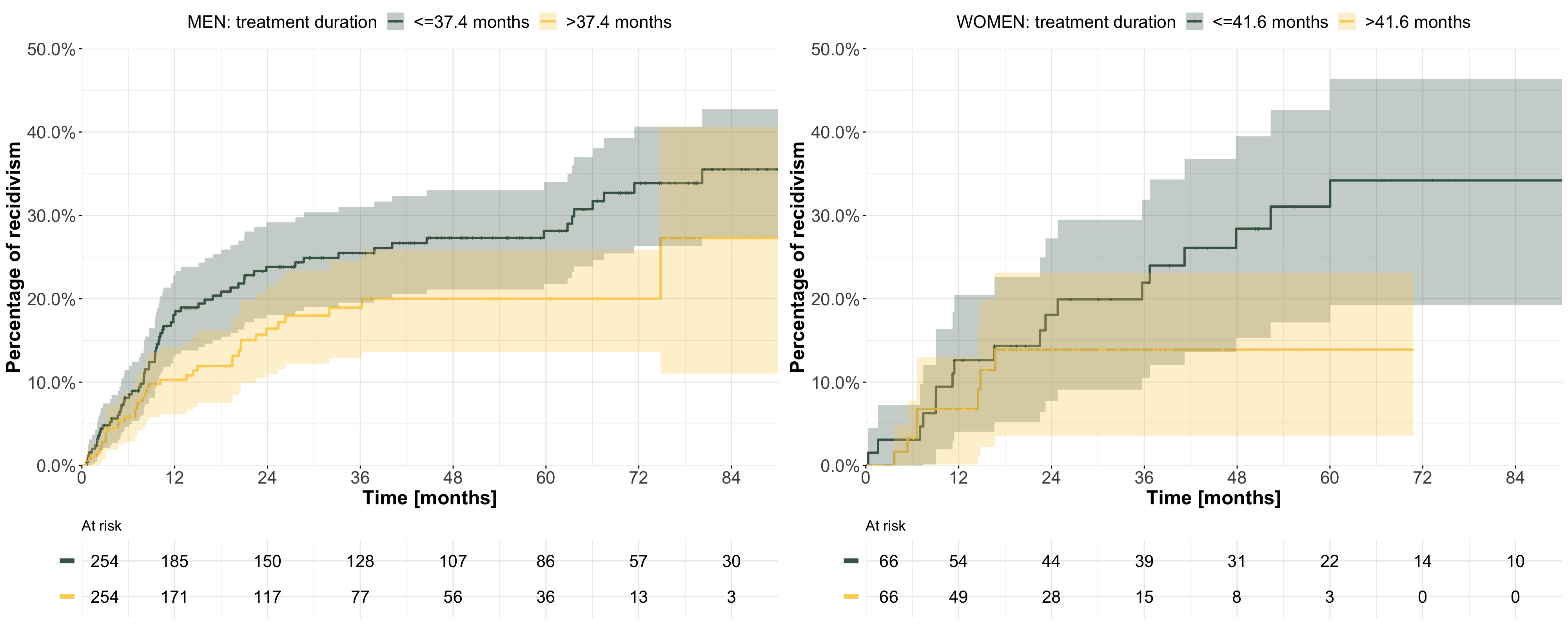

Supplement: SUPPLEMENTARY FIGURE 4 — Estimated time to reoffending after discharge from forensic psychiatric care after stratification by sex and dichotomization according to treatment duration. [file Image_4.JPEG]

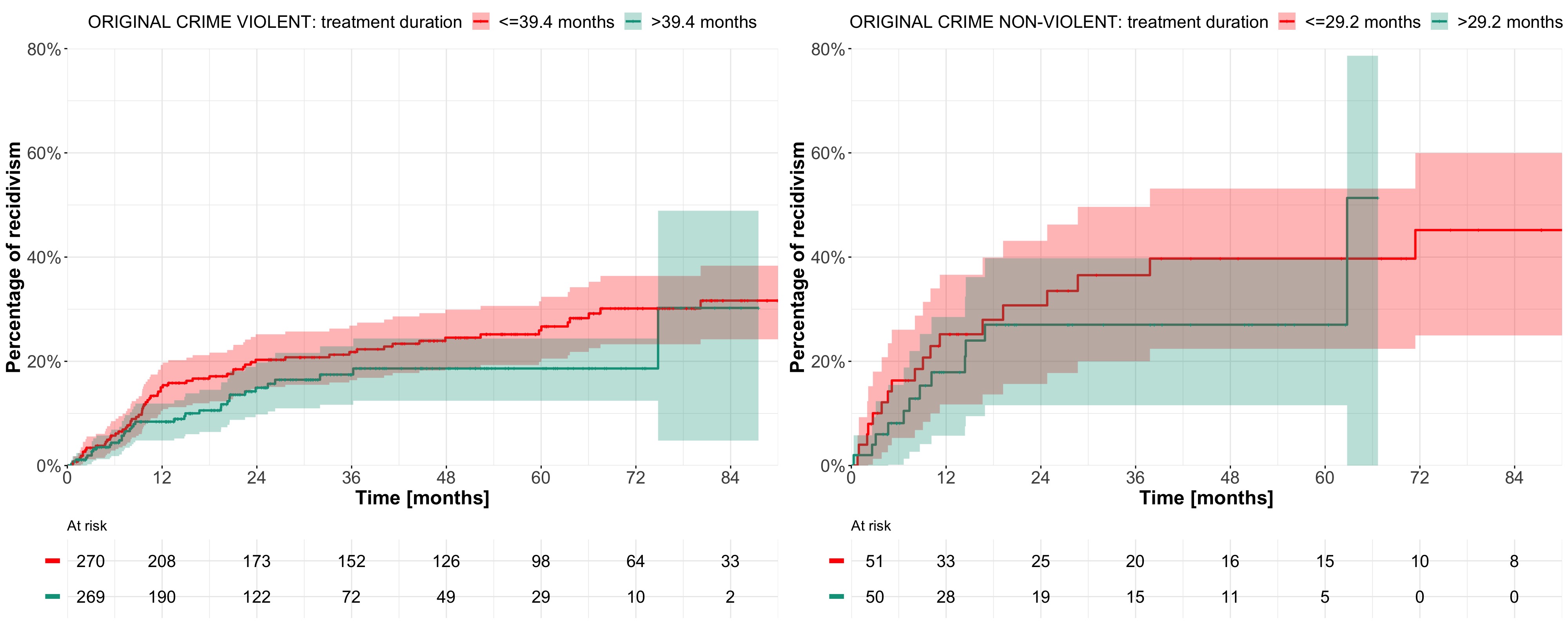

Supplement: SUPPLEMENTARY FIGURE 5 — Estimated time to reoffending after discharge from forensic psychiatric care after stratification by original crime and dichotomization according to treatment duration. [file Image_5.JPEG]

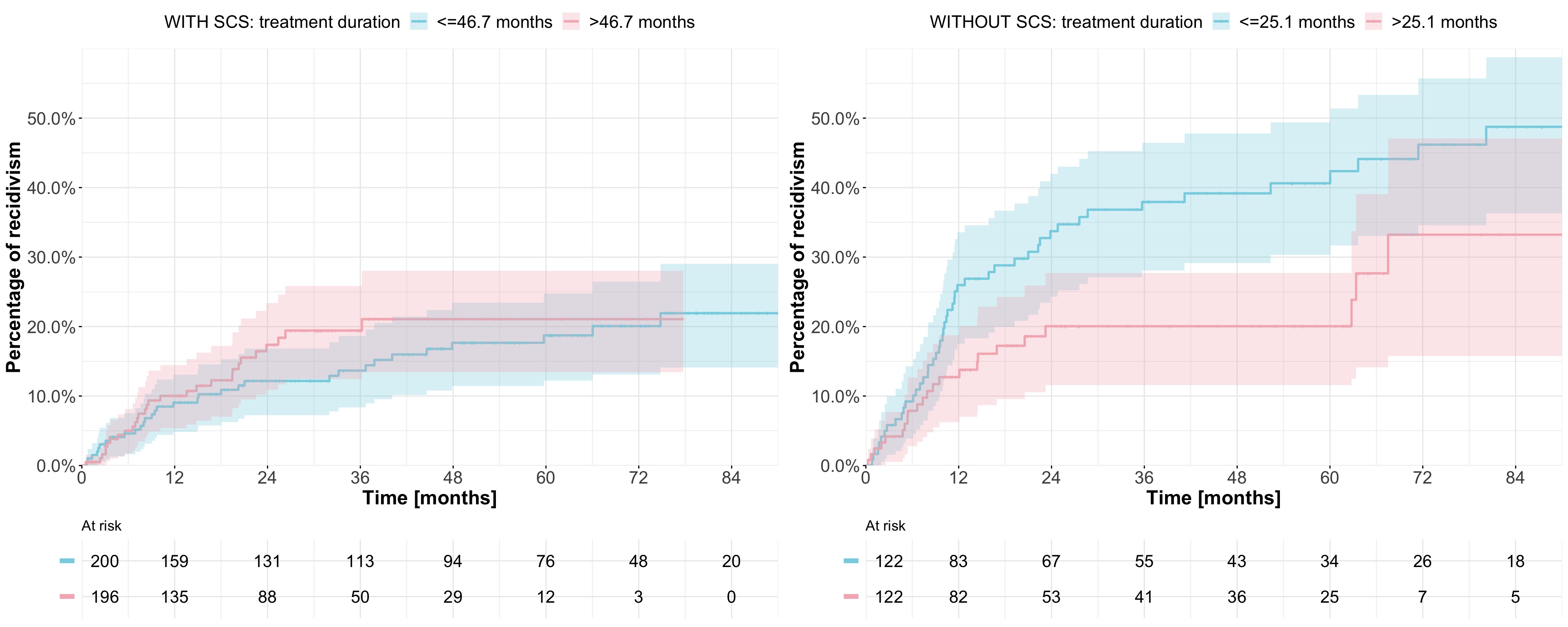

Supplement: SUPPLEMENTARY FIGURE 6 — Estimated time to reoffending after discharge from forensic psychiatric care after stratification by sentence type and dichotomization according to treatment duration. SCS = special court supervision. [file Image_6.JPEG]

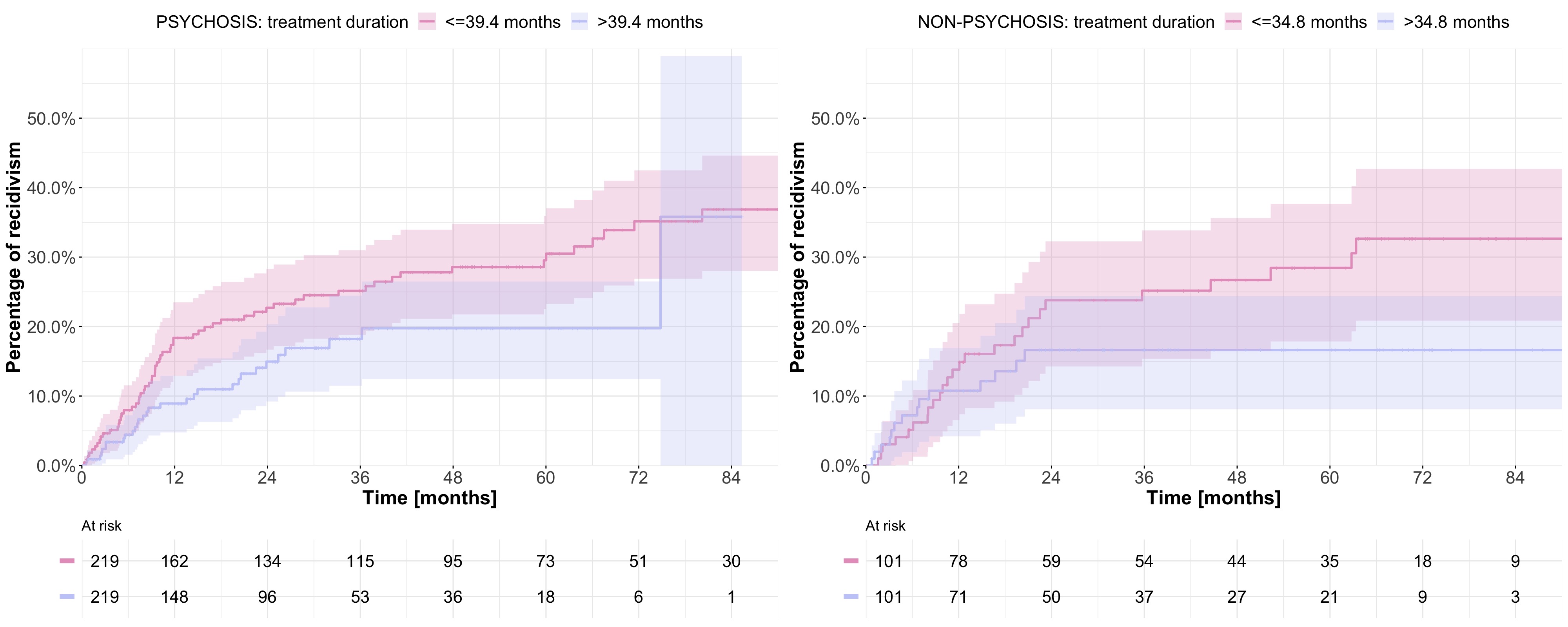

Supplement: SUPPLEMENTARY FIGURE 7 — Estimated time to reoffending after discharge from forensic psychiatric care after stratification by diagnosis and dichotomization according to treatment duration. [file Image_7.JPEG]

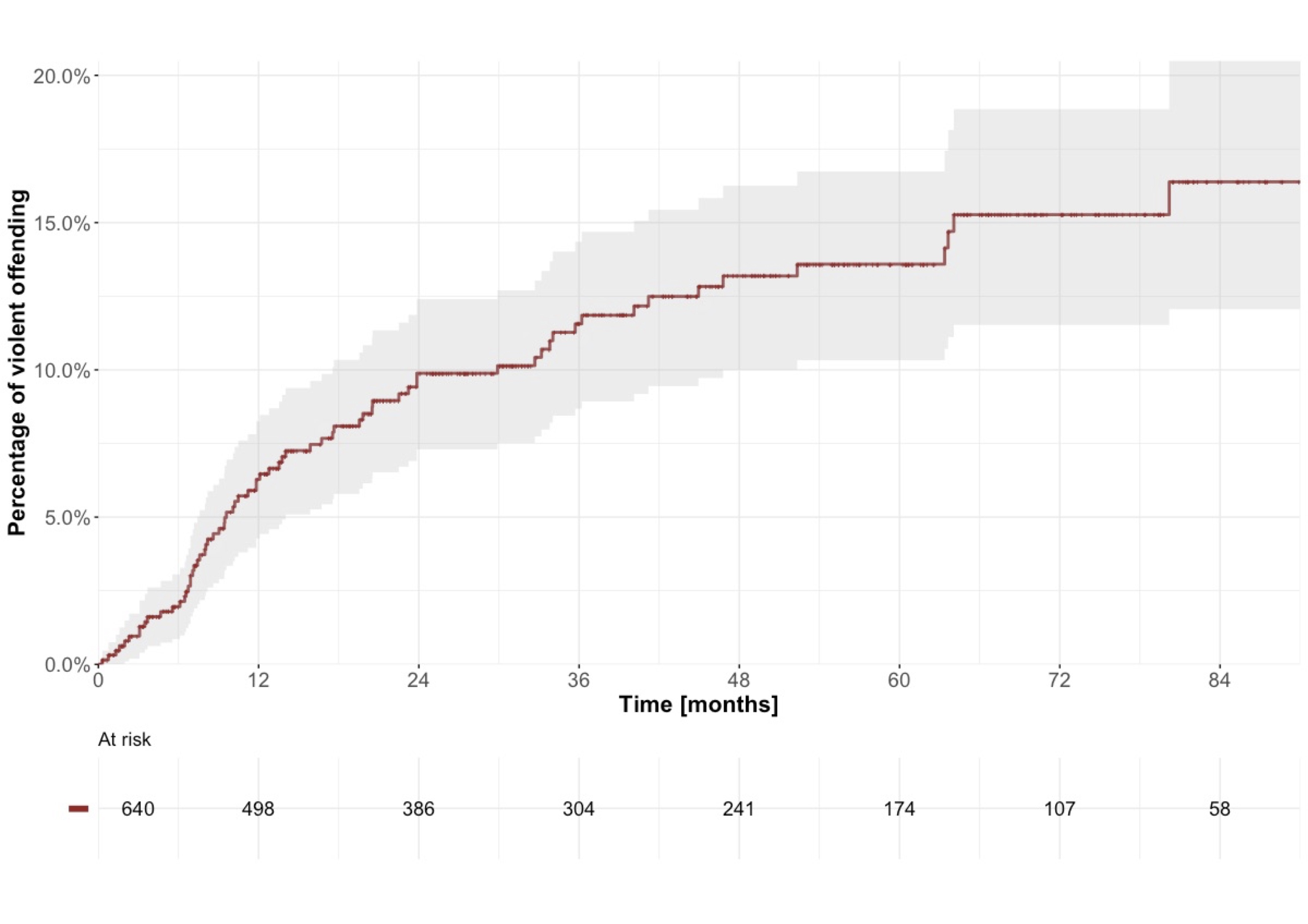

Supplement: SUPPLEMENTARY FIGURE 8 — Estimated time to violent offending after discharge from forensic psychiatric care. [file Image_8.JPEG]

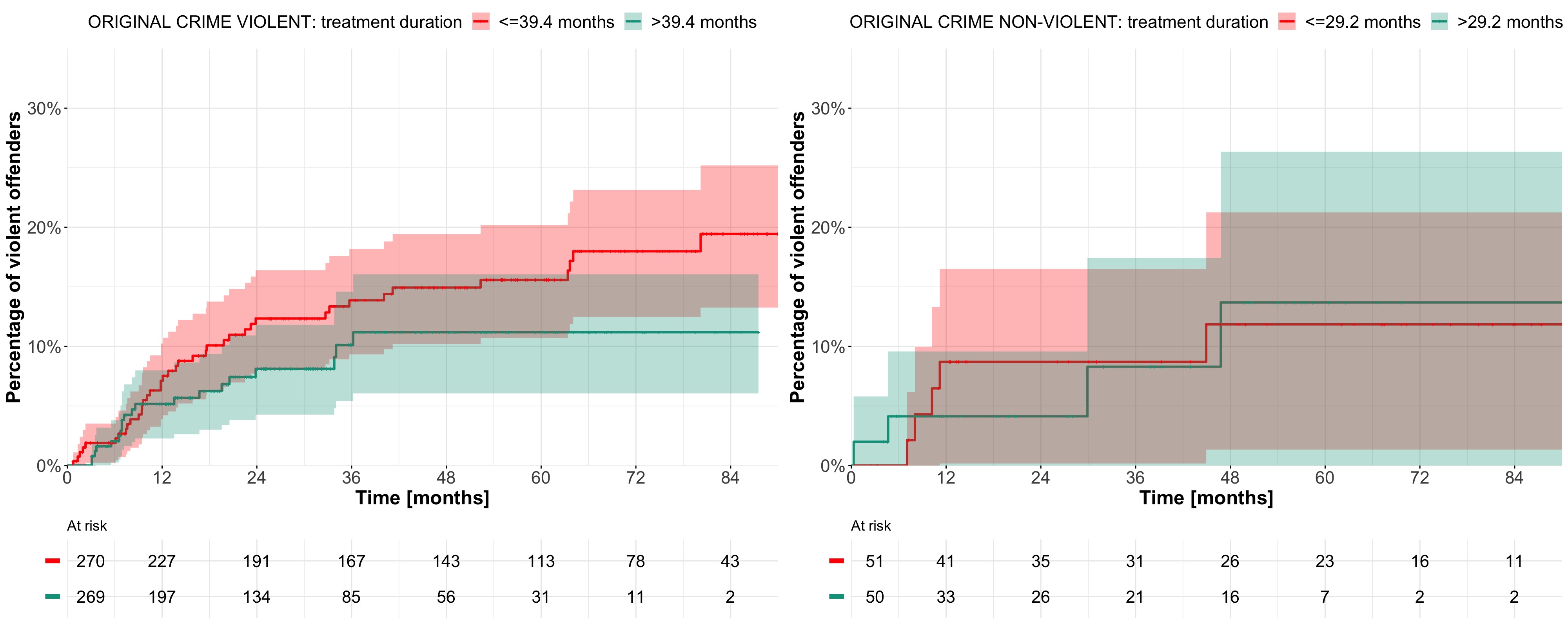

Supplement: SUPPLEMENTARY FIGURE 9 — Estimated time to violent offending after discharge from forensic psychiatric care after stratification by original crime and dichotomization according to treatment duration. [file Image_9.JPEG]

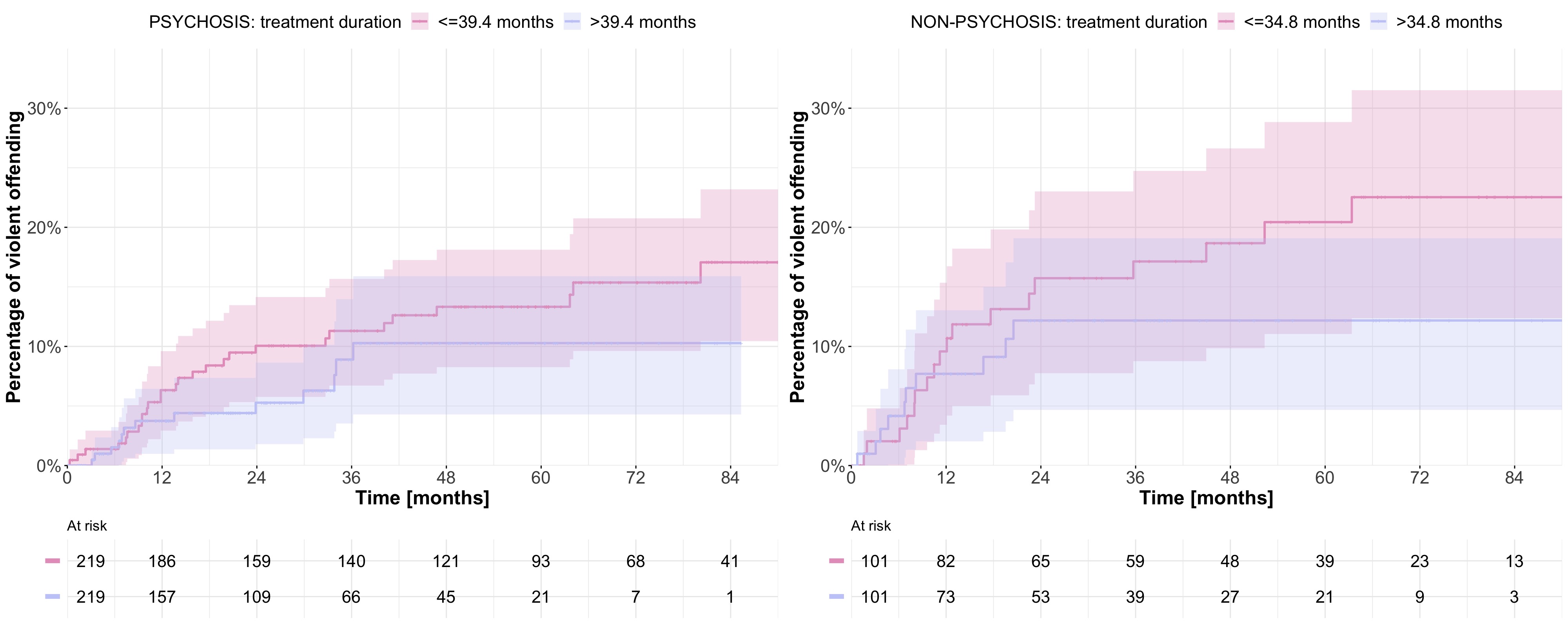

Supplement: SUPPLEMENTARY FIGURE 10 — Estimated time to violent offending after discharge from forensic psychiatric care after stratification by diagnosis and dichotomization according to treatment duration. [file Image_10.JPEG]

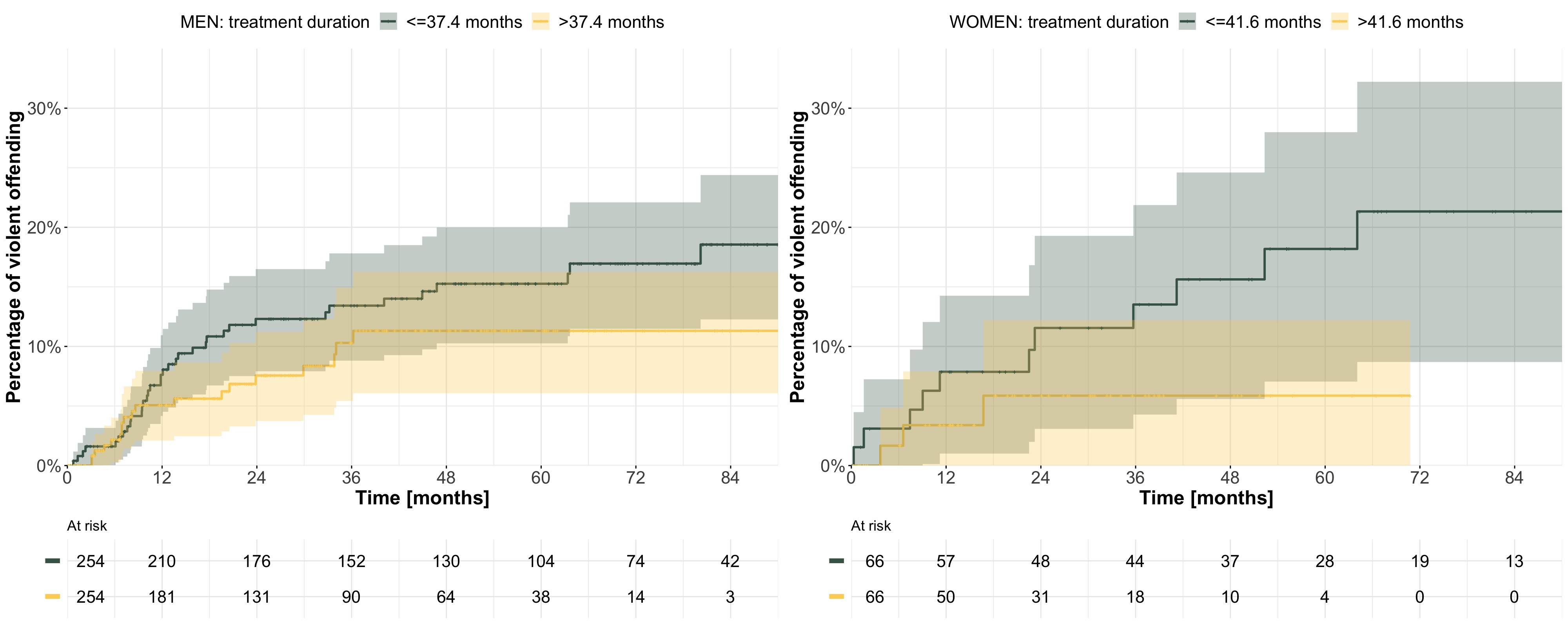

Supplement: SUPPLEMENTARY FIGURE 11 — Estimated time to violent offending after discharge from forensic psychiatric care after stratification by sex and dichotomization according to treatment duration. [file Image_11.JPEG]

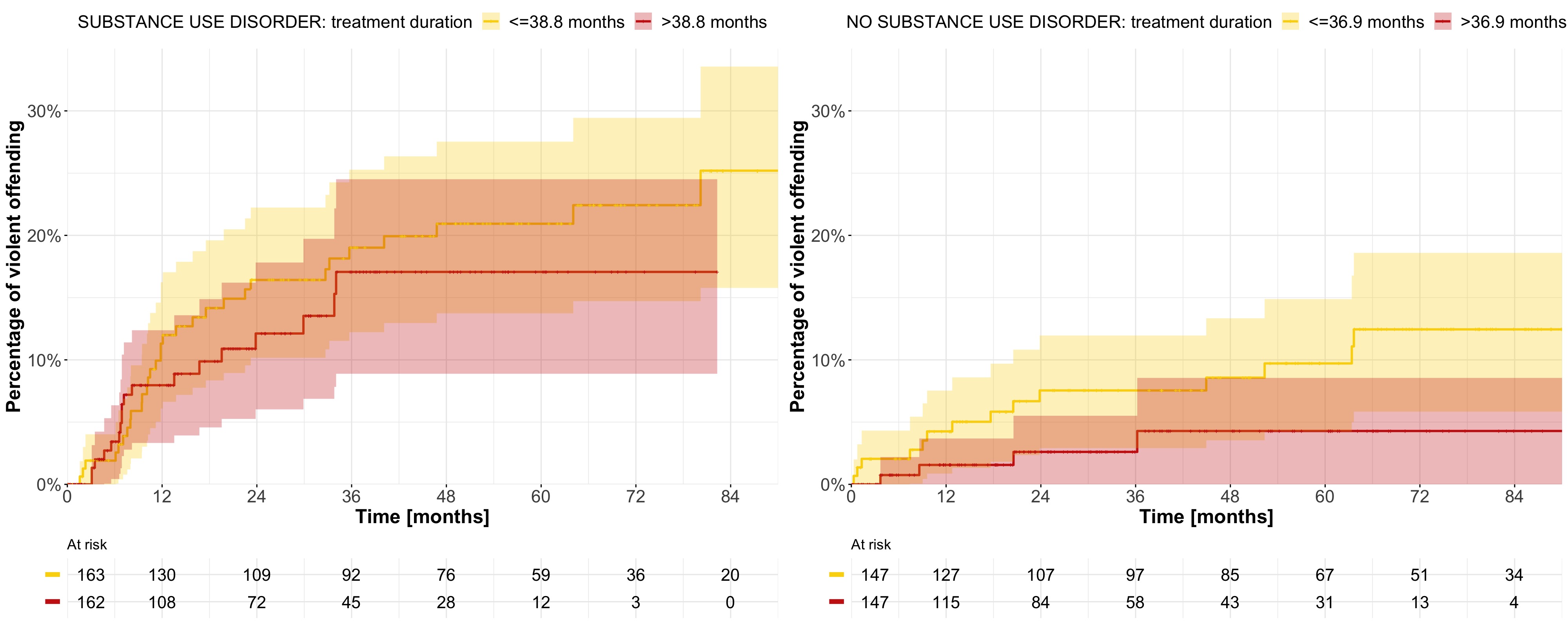

Supplement: SUPPLEMENTARY FIGURE 12 — Estimated time to violent offending after discharge from forensic psychiatric care after stratification by history of substance use disorder and dichotomization according to treatment duration. [file Image_12.JPEG]

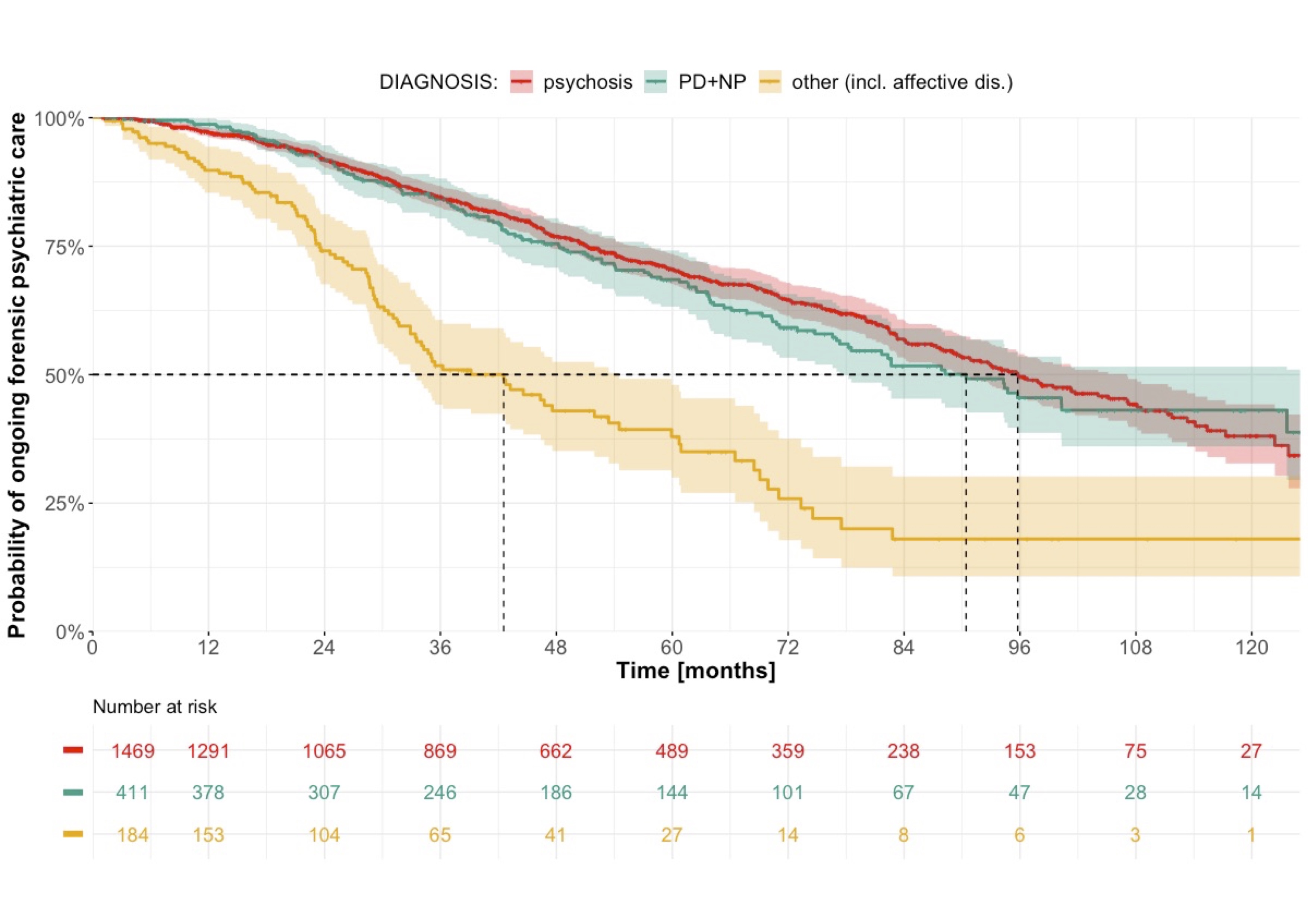

Supplement: SUPPLEMENTARY FIGURE 13 — Estimated time from sentence to discharge from forensic psychiatric care with regard to diagnosis (alternative coding). PD + NP = developmental and personality disorders. [file Image_13.JPEG]

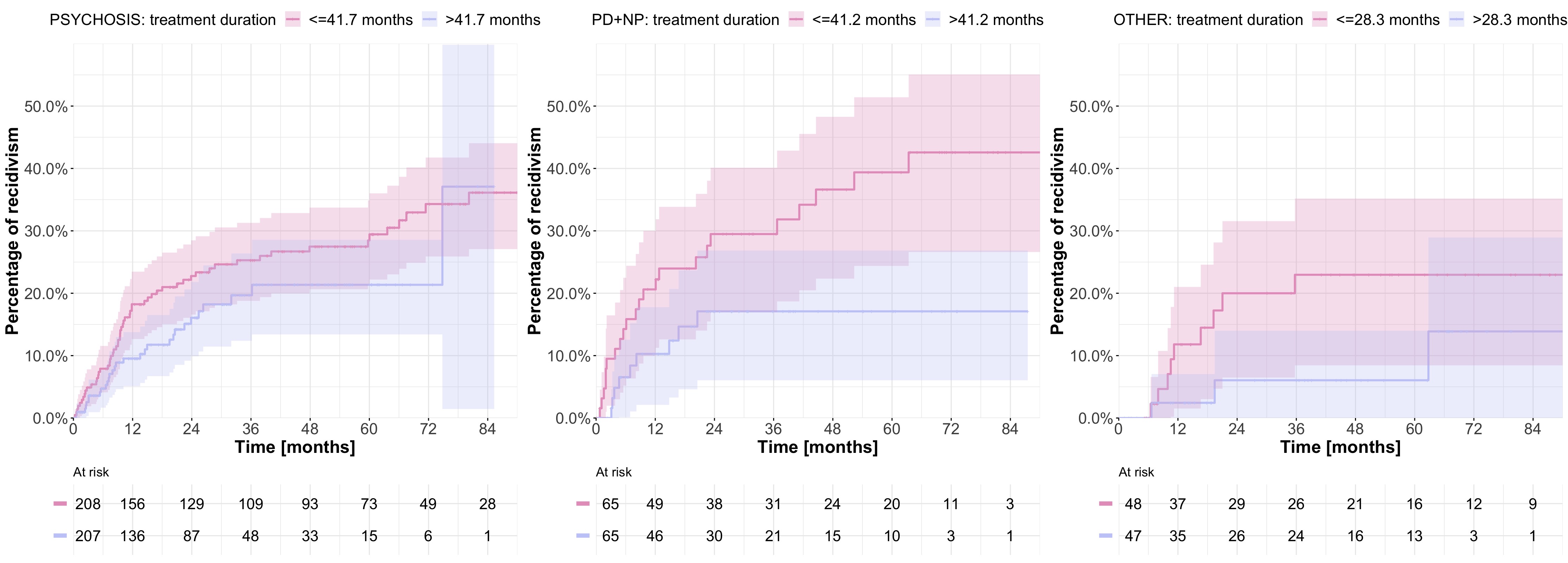

Supplement: SUPPLEMENTARY FIGURE 14 — Estimated time to reoffending after discharge from forensic psychiatric care after stratification by diagnosis (alternative coding) and dichotomization according to treatment duration. PD + NP = developmental and personality disorders. [file Image_14.JPEG]

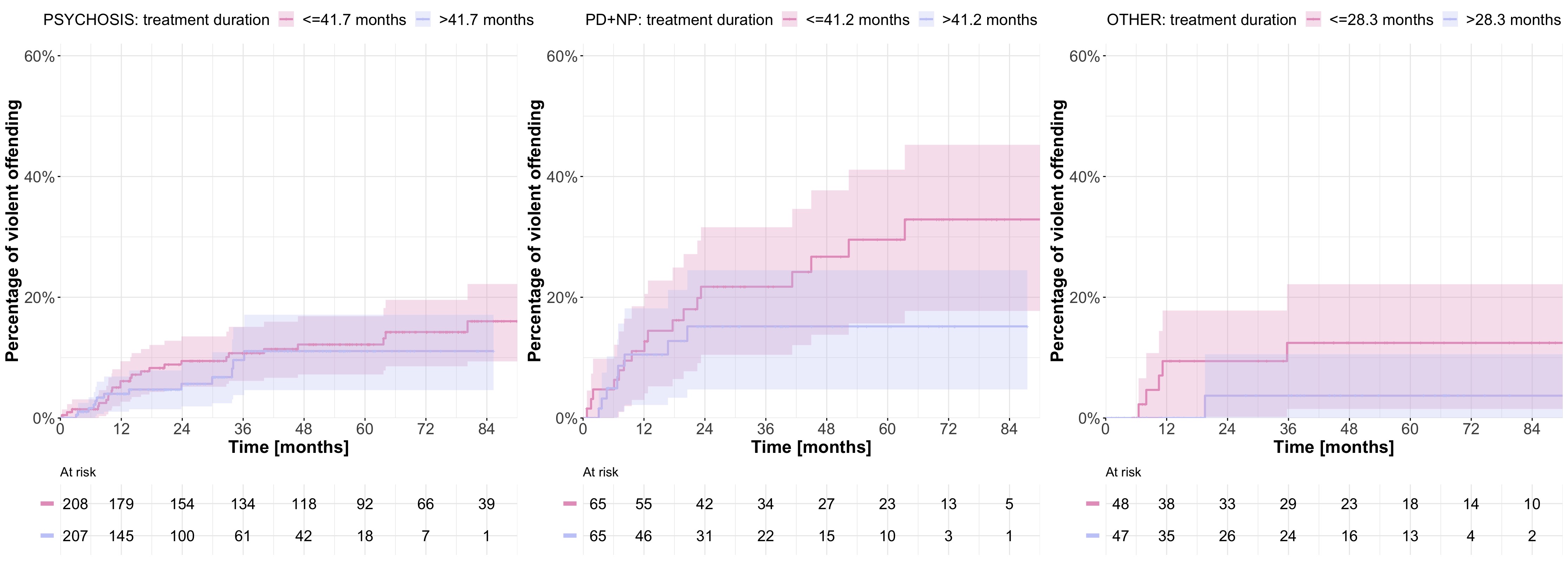

Supplement: SUPPLEMENTARY FIGURE 15 — Estimated time to violent offending after discharge from forensic psychiatric care after stratification by diagnosis (alternative coding) and dichotomization according to treatment duration. PD + NP = developmental and personality disorders. [file Image_15.JPEG]
